# Integrin but not CEACAM receptors are dispensable for *Helicobacter pylori* CagA translocation

**DOI:** 10.1371/journal.ppat.1007359

**Published:** 2018-10-26

**Authors:** Qing Zhao, Benjamin Busch, Luisa Fernanda Jiménez-Soto, Hellen Ishikawa-Ankerhold, Steffen Massberg, Laurent Terradot, Wolfgang Fischer, Rainer Haas

**Affiliations:** 1 Chair of Medical Microbiology and Hospital Epidemiology, Max von Pettenkofer Institute, Faculty of Medicine, LMU Munich, Germany; 2 Medizinische Klinik und Poliklinik I, Ludwig-Maximilians-Universität, Munich, Germany; 3 UMR 5086 Molecular Microbiology and Structural Biochemistry, Institut de Biologie et Chimie des Protéines, CNRS-Université de Lyon, France; 4 German Center for Infection Research (DZIF), Munich Site, Munich, Germany; University of Illinois, UNITED STATES

## Abstract

Translocation of the *Helicobacter pylori* (*Hp*) cytotoxin-associated gene A (CagA) effector protein via the *cag*-Type IV Secretion System (*cag*-T4SS) into host cells is a hallmark of infection with *Hp* and a major risk factor for severe gastric diseases, including gastric cancer. To mediate the injection of CagA, *Hp* uses a membrane-embedded syringe-like molecular apparatus extended by an external pilus-like rod structure that binds host cell surface integrin heterodimers. It is still largely unclear how the interaction of the *cag*-T4SS finally mediates translocation of the CagA protein into the cell cytoplasm. Recently certain carcinoembryonic antigen-related cell adhesion molecules (CEACAMs), acting as receptor for the *Hp* outer membrane adhesin HopQ, have been identified to be involved in the process of CagA host cell injection. Here, we applied the CRISPR/Cas9-knockout technology to generate defined human gastric AGS and KatoIII integrin knockout cell lines. Although confocal laser scanning microscopy revealed a co-localization of *Hp* and β1 integrin heterodimers on gastric epithelial cells, *Hp* infection studies using the quantitative and highly sensitive *Hp* β-lactamase reporter system clearly show that neither β1 integrin heterodimers (α1β1, α2β1 or α5β1), nor any other αβ integrin heterodimers on the cell surface are essential for CagA translocation. In contrast, deletion of the HopQ adhesin in *Hp*, or the simultaneous knockout of the receptors CEACAM1, CEACAM5 and CEACAM6 in KatoIII cells abolished CagA injection nearly completely, although bacterial binding was only reduced to 50%. These data provide genetic evidence that the *cag*-T4SS-mediated interaction of *Hp* with cell surface integrins on human gastric epithelial cells is not essential for CagA translocation, but interaction of *Hp* with CEACAM receptors is facilitating CagA translocation by the *cag-*T4SS of this important microbe.

## Author summary

The Cag Type IV secretion system of *Helicobacter pylori* (*Hp*) interacts with host cell integrins and injects the bacterial oncoprotein CagA into host cells thereby contributing to inflammation and carcinogenesis during chronic infection. Binding of β1 integrin receptors by the CagA protein and the type IV secretion system is well described by many research groups, but its function for CagA translocation is not well understood. We report here that this interaction is not essential for the function of the secretion system and for CagA injection into the gastric epithelial cells lines AGS and KatoIII. However, the bacterial binding to a set of specific receptors called carcinoembryonic antigen-related cell adhesion molecules (CEACAMs) by the *Hp* outer membrane protein HopQ is a prerequisite for CagA translocation. Interestingly, other bacterial adhesins and the mediated binding events do not have a similar effect on CagA translocation, suggesting a specific feature associated with HopQ mediated binding.

## Introduction

Secretion systems of Gram-negative bacteria have evolved to mediate the passage of macromolecules across two or more cellular membranes, either into the extracellular space, or directly into selected host target cells [1]. A highly versatile group represents the bacterial Type IV secretion systems (T4SS), which can transport diverse components in a contact-dependent manner, ranging from single proteins to protein-protein and protein-DNA complexes [2, 3]. One of these bacteria is *Helicobacter pylori* (*Hp*), which is recognized as one of the most prevalent bacterial pathogens worldwide and very efficiently utilizes the cytotoxin-associated gene (*cag*) type IV secretion system (*cag*-T4SS) as a major virulence determinant [4, 5]. The effector protein CagA, together with a set of 27 proteins acting as structural and/or regulatory elements of the T4SS, are encoded on the *cag* pathogenicity island (*cag*PAI), approximately 40 kb in size. Upon host cell contact the *cag*-T4SS forms needle-like surface appendages, the T4SS pili [6–8], which are involved in the translocation of CagA from cell-adherent *Hp* across the bacterial and epithelial membranes into the host cell cytoplasm [8]. Our view on these fascinating nanomachines was extended recently by ultrastructural insights into the *cag*-T4SS-dependent membranous pilus-like appendages by *in vivo* electron cryotomography [9]. Injected CagA is tyrosine-phosphorylated on multiple Glu-Pro-Ile-Tyr-Ala (EPIYA) motifs in the C-terminal region, allowing its interaction with a set of cellular target proteins [10, 11]. This results in dysregulation of the homeostatic signal transduction events in gastric epithelial cells, in loss of cell polarity, chronic inflammation and malignancy, qualifying CagA as a bacterial oncoprotein [12].

The *cag*-T4SS targets host cells via β1 integrin receptors [13, 14], and induces in these cells the production and secretion of proinflammatory cytokines and chemokines, such as interleukin-8 (IL-8) [15]. The pilus-associated protein CagL has originally been reported to interact via an arginine-glycine-aspartate (RGD) motif with the α5β1 integrin heterodimer and thereby to activate Src and focal adhesion kinase, however, the requirement of the RGD motif for T4SS functionality was assessed differently [13, 14]. Other *cag*PAI proteins, including CagY, CagI and CagA, have also been identified as interacting with the α5β1 integrin and an integrin binding domain for CagA was identified [16, 17].

In addition to receptor binding by the *cag*T4SS itself, the outer membrane protein HopQ was identified to support CagA translocation by acting as a non-*cag*PAI-encoded cofactor of T4SS function [18]. Later on, HopQ was found to selectively bind a set of receptors from the carcinoembryonic antigen-related cell adhesion molecule family (CEACAMs). CEACAM1, CEACAM3, CEACAM5 and CEACAM6 were identified as functional receptors for *Hp* via the outer membrane protein HopQ [19, 20]. *Hp*-CEACAM binding not only plays a role for *Hp* adherence, but this interaction deeply contributes to the process of CagA translocation. Thus, the human embryonic kidney cell line (HEK293), which is devoid of CEACAM receptors on its surface, was resistant for CagA injection by *Hp*, but became readily susceptible upon functional expression of CEACAM1 or CEACAM5 on its surface [19, 20].

The purpose of this study was to further dissect the role of integrin receptors versus the function of CEACAM receptors for the *cag*T4SS in the process of CagA translocation. Using the CRISPR/Cas9 system, we systematically generated single to multiple integrin knockout epithelial human cell lines (AGS and KatoIII) ending up with KatoIII cells without any integrin heterodimers on their surface. Unexpectedly, CagA translocation into these completely integrin-deficient cells was not significantly changed, suggesting that other integrin-independent *Hp*–host cell interactions must be important. In contrast, CRISPR/Cas9-mediated knockout of CEACAM receptors (CEACAM1, CEACAM5 and CEACAM6 simultaneously) generated in KatoIII cells resulted in a strong reduction of CagA translocation capacity by *Hp*, suggesting that β-integrin receptors play a minor role in the T4SS-mediated CagA translocation, but the *Hp-*CEACAM interaction is of major importance.

## Results

### Human gastric AGS and KatoIII cells produce a similar set of integrin heterodimers on their cell surface

**Fig 1:**
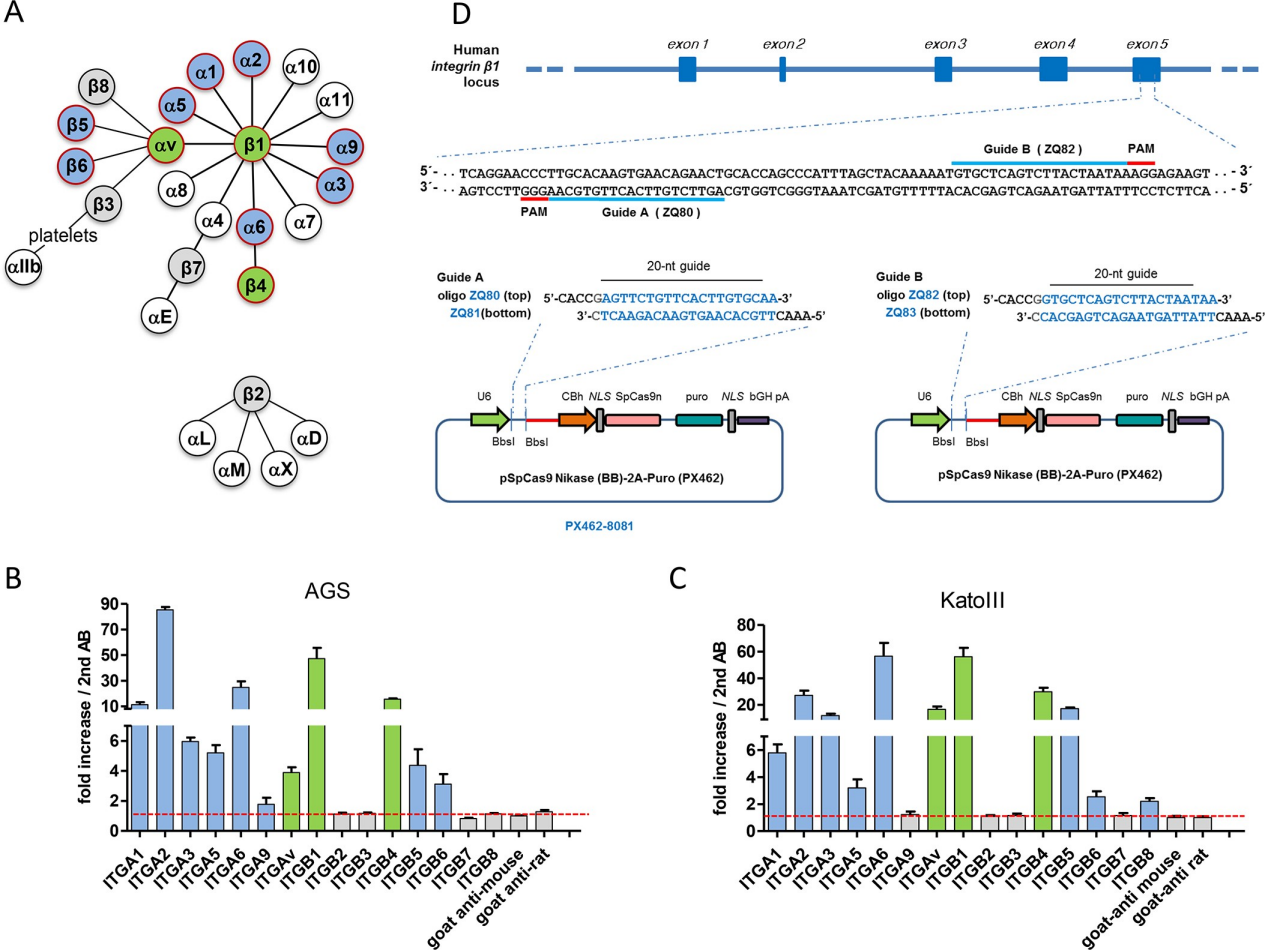
Schematic representation of the mammalian integrin receptor family, integrin profiling in AGS and KatoIII gastric cell lines and the strategy for integrin β1 knockout generation. **A)** Illustration of possible integrin α and β associations [21]. Epithelial cell-specific heterodimers are marked with red circles, α and β subunits expressed in AGS or KatoIII cells, as determined in **B** and **C**, are shown as filled blue or green (integrin genes targeted by CRISPR/Cas-mediated gene knockout) circles. Grey and white circles represent subunits tested but not expressed, or not tested for expression, respectively. **B)** Integrin expression profile of AGS cells as determined by flow cytometry using different integrin antibodies. **C**) Integrin expression profile of KatoIII cells as determined by flow cytometry. **D**) Strategy for targeted deletion of integrin β1 gene. *Streptococcus pyogenes* Cas9 nickase binding sites (20 bp, highlighted in blue) are immediately followed by the 5’-NGG PAM (protospacer adjacent motif). The short guide RNA (sgRNA) pairs are located on both strands of the target DNA with a 25 bp gap. Cloning scheme of the CRISPR plasmids (see Materials and methods for details). All values in **B** and **C** were determined as standard errors of the mean (±SEM) from three independent experiments.

The integrin receptor family is composed of 24 distinct integrin heterodimers, generated by different α and β subunits. Generally, integrin receptors follow a distinct tissue- and cell type-specific expression pattern in epithelial cells, leukocytes or platelets [21]. Thus, six β1 integrin heterodimers (α1β1, α2β1, α3β1, α5β1, α6β1 and α9β1), two αv integrins (αvβ5 and αvβ6) and the integrin α6β4 are known to be epithelial-specific (Fig 1A) [21].

AGS and KatoIII cell lines are generally used as model systems for the evaluation of CagA translocation, since both cell lines were derived from human gastric epithelial cells. To get an overview of integrin expression on the surface of these cells, we stained them with different integrin-specific antibodies and determined the integrin expression profile by flow cytometry. AGS and KatoIII cells indeed produced β1 integrins (including α1β1, α2β1, α3β1, α5β1, α6β1 and α9β1), αv integrins (αvβ5 and αvβ6) (αvβ8 only by KatoIII) and the β4 integrin (α6β4) on their surface, however with varying expression levels (Fig 1B and 1C).

We planned to generate a β1 gene knockout in AGS cells that should lack surface expression of all potential β1 containing integrins, since the targeting of either subunit of a given integrin heterodimer should ultimately result in the depletion of the targeted integrin heterodimer [22, 23]. In order to obtain integrin knockout cell lines without undesired off-target mutagenesis, the double nicking strategy was applied [24]. For design of paired short guide RNAs (sgRNAs) targeting the integrin β1 (ITGB1) gene, the online CRISPR design tool (http://tools.genome-engineering.org) was used for optimal sgRNA analysis and identification (See further details of the method in Experimental Procedures) (Fig 1D and S1 Table).

### AGS cells devoid of surface α/β1 integrin heterodimers are fully competent for CagA translocation

**Fig 2:**
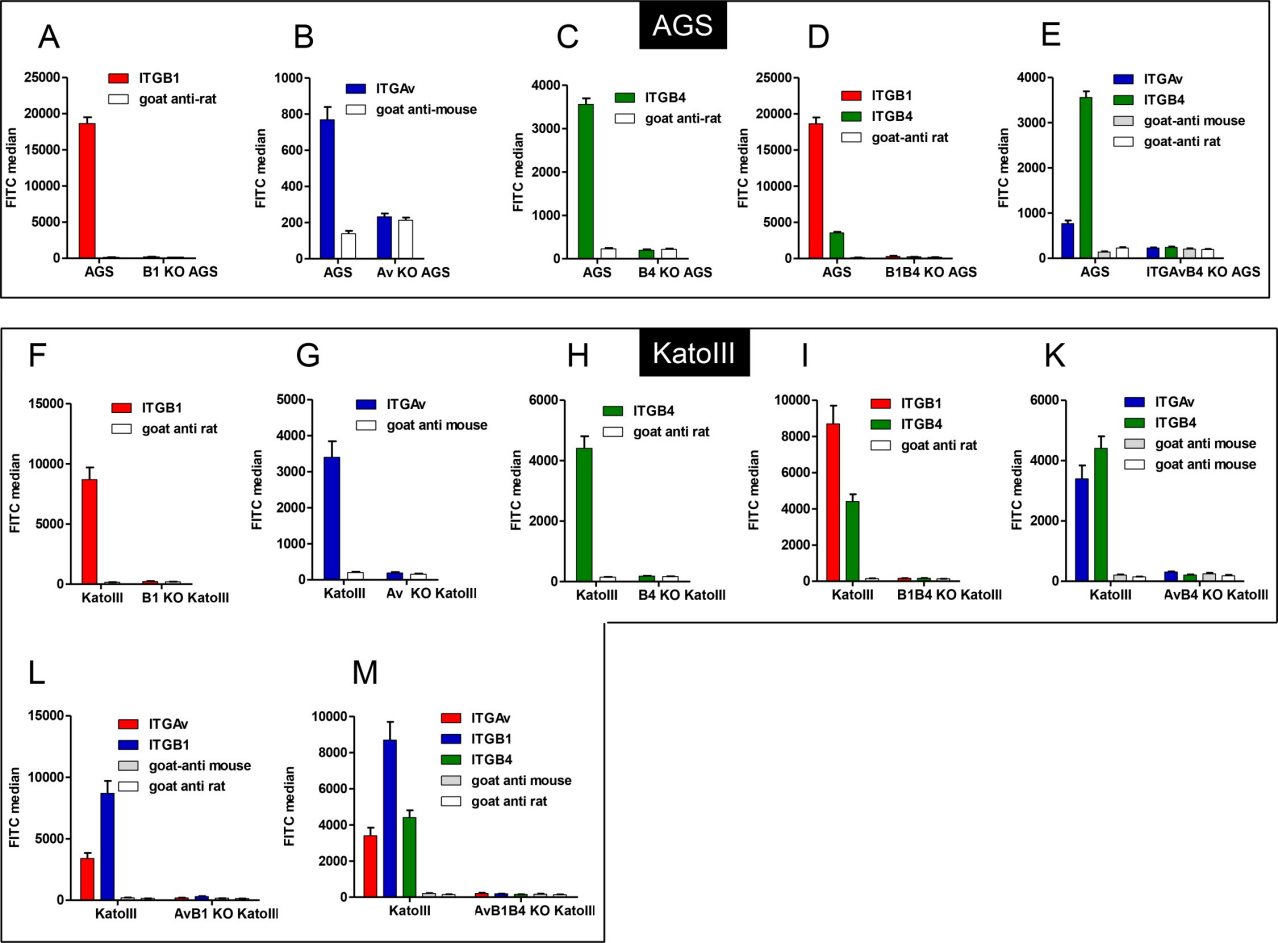
Integrin expression of AGS and KatoIII wild type and corresponding single and multiple integrin-knockout cell lines. **A-E)** Integrin expression was determined showing FITC median from three independent flow cytometry experiments. As negative controls, cells were stained with secondary antibody only (Goat-anti mouse, Goat-anti rat). **A**) ITGB1 surface expression in wild type and ITGB1 KO AGS cells. **B**) ITGAv surface expression in wild type and ITGAv KO AGS cells. **C**) ITGB4 surface expression in wild type and ITGB4 KO AGS cells. **D**) ITGB1 and ITGB4 surface expression in wild type and ITGB1B4 KO AGS cells. **E**) ITGAv and ITGB4 surface expression in wild type and ITGAvB4 KO AGS cells. **F-M)** Integrin expression was determined showing FITC median from three independent flow cytometry experiments. As negative controls, cells were stained with secondary antibody only (Goat-anti mouse, Goat-anti rat). **F**) ITGB1 surface expression in wild type and ITGB1 KO KatoIII cells. **G**) ITGAv surface expression in wild type and ITGAv KO KatoIII cells. **H**) ITGB4 surface expression in wild type and ITGB4 KO KatoIII cells. **I**) ITGAv and ITGB1 surface expression in wild type and ITGAvB1 KO KatoIII cells. **K**) ITGAv and ITGB4 surface expression in wild type and ITGAvB4 KO KatoIII cells. **L**) ITGB1 and ITGB4 surface expression in wild type and ITGB1B4 KO KatoIII cells. **M**) ITGAv, ITGB1 and ITGB4 surface expression in wild type and ITGB1AvB4 KO KatoIII cells. All values are indicated as average values including standard errors of the mean (±SEM), (n = 3).

For generation of a β1 integrin deficient AGS cell line, verified CRISPR constructs targeting exon 5 of the β1 integrin gene (ITGB1) were transfected into AGS cells. Transfected cells went through a selection procedure to obtain knockout cell lines. Since CRISPR constructs contain the puromycin resistance gene, the transfected population was treated with puromycin to kill non-transfected cells. The surviving cells were stained with integrin β1 antibody for negative selection by FACS sorting. Finally, serial dilutions of the sorted negative populations resulted in stable cell lines, which could be verified as completely integrin β1-deficient by flow cytometry analysis (Fig 2A). Furthermore, the complete absence of the gene product was verified by (i) demonstrating the disruption of the targeted gene sequence by PCR amplification and sequencing of the integrin β1 alleles (S1A Fig) and (ii) by immunoblotting of cell lysates using a β1 integrin-specific antibody (S2A Fig).

**Fig 3:**
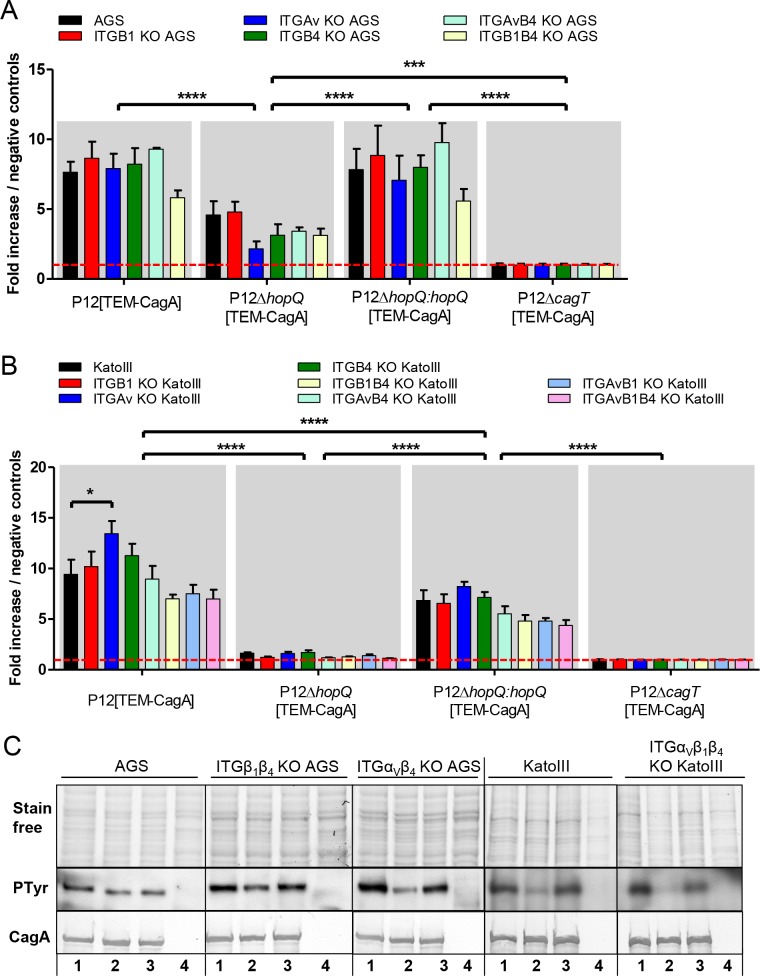
CagA tyrosine phosphorylation and quantitative evaluation of CagA translocation into wild type integrin-knockout AGS or KatoIII cell lines by the TEM-1 β-lactamase reporter assay. **A)** AGS and five integrin-depletion cell lines in 96-well plates were infected with P12[TEM-CagA], the P12Δ*hopQ*[TEM-CagA], the genetically complemented P12Δ*hopQ*:*hopQ*[TEM-CagA] and as negative control the translocation-deficient P12Δ*cagT*[TEM-CagA] deletion mutant at an MOI of 60. **B)** KatoIII wild type and seven single or multiple integrin depletion cell lines in 96-well plates were infected with P12[TEM-CagA], the P12Δ*hopQ*[TEM-CagA], the genetically complemented P12ΔhopQ:hopQ[TEM-CagA] and as negative control the translocation-deficient P12Δ*cagT*[TEM-CagA] deletion mutant at an MOI of 60. Ratios of blue to green fluorescence of each sample were calculated and normalized to the mean of blue to green ratio of the negative controls. All values were indicated as standard errors of the mean (±SEM) from n = 3 independent experiment for AGS and n = 5 independent experiments for KatoIII cells. The red line marks the level of the controls. **C)** AGS cells, two AGS integrin-depletion cell lines (ITGB1B4 KO, ITGAvB4 KO), KatoIII cells and the triple integrin-depletion KatoIII cell line (ITGAvB1B4 KO) were infected with strain P12, P12Δ*hopQ* or P12Δ*hopQ*:*hopQ*, for 2.5 hours with an MOI of 60. Translocation of CagA was determined by detecting tyrosine-phosphorylated CagA with the anti phosphotyrosine antibody PY99. Statistics: Two-way ANOVA with a Tukey’s multiple comparison Post-Hoc test was performed. (* P < 0.05; ** P < 0.01, *** P < 0.001, **** P < 0.0001). Values within each group did not change significantly, with exception of KatoIII wild type versus ITGAv KO KatoIII cells infected by P12 [TEM-CagA].

Next, the verified β1 integrin-deficient AGS cells were tested for CagA translocation capacity by *Hp*. Traditionally, CagA translocation is assessed by detecting tyrosine-phosphorylated EPIYA motifs as a phosphorylated CagA band via western blot. This can be used for quantification, but is not very sensitive and accurate. We have recently established a sensitive β-lactamase reporter system (TEM-1 reporter assay) to accurately determine *Hp* CagA translocation into host cells independently of its tyrosine phosphorylation and host cell kinase activity [25]. When applying the *Hp* strain P12[TEM-CagA] in the TEM-1 reporter assay, we surprisingly did not observe a significant difference in CagA translocation into AGS wild type versus β1 integrin-deficient cells (Fig 3A). This observation suggested that the β1-integrin interaction was apparently not essential for the bacteria or the *cag*-T4SS to inject CagA.

### Double knockout AGS cells (ITGAvB4, ITGB1B4) translocate CagA efficiently

One possible explanation for this unexpected result might be that other integrin heterodimers (αvβ5, αvβ6, or α6β4), which are known to be expressed on AGS cells (see Fig 1A), are able to functionally substitute β1 integrin heterodimers regarding CagA translocation. We therefore extended the CRISPR/Cas9-mediated knockout strategy to inactivate integrins αv and β4 separately, using the same procedure as for integrin β1 (see S3 Fig and S4 Fig for design of integrin gene inactivation strategy). Furthermore, by targeting different combinations of two of the aforementioned genes in the same cell, different combinations of double mutants were obtained (ΔITGAvB4, ΔITGB1B4), which did not produce the corresponding integrins on the cell surface, as determined by flow cytometry (Fig 2B–2E). A concomitant knockout of all three integrin genes, which ultimately should result in cell lines devoid of all integrins on the AGS cell surface, could not be obtained in the AGS cell background, probably because they do not survive.

The correct genetic inactivation of the integrin genes was verified by PCR amplification and sequencing of the corresponding αv- or β4-specific integrin alleles (S1B and S1C Fig). The complete absence of the gene products was confirmed by immunoblotting with integrin αv- or β4-specific antibodies (S2A Fig). We next asked whether obvious differences in the morphology, physiology or function of the integrin-knockout AGS derivatives are apparent compared to wild type AGS cells. A thorough study of the general cell morphology did not show any peculiarities. AGS AvB4 cells did only grow in tissue culture when collagen was added, which indicated that the integrin receptor-mediated binding to certain integrin ligands was absent. All wild type and knockout mutant cells showed the hummingbird phenotype (S5 Fig). IL-8 induction in AGS wild type cells was slightly reduced in the P12Δ*hopQ* infecting strain, as compared to the P12 wild type (wt) and the complemented mutant strain (S6 Fig). Interestingly the level of IL-8 induction was generally higher when integrin knockout cells were used as compared to AGS wild type cells, but the general pattern of reaction of the knockout versus the wild type cells was well conserved (S6 Fig). This indicates that the main phenotypic characteristics of the knockout cells are still conserved in comparison to wild-type cells, arguing against unexpected compensatory mutations or significant alterations in signal transduction networks in the knockout cells.

Interestingly, infection experiments based on the TEM-1 reporter assay showed no statistically significant difference in CagA translocation efficiency into AGS wild type versus single or multiple integrin αv- or β deficient cells (Fig 3A). Similar results were obtained by the conventional tyrosine phosphorylation experiments upon infection of the mutant AGS epithelial cell lines (Fig 3C).

In conclusion, we demonstrate here that *Hp* is able to translocate its CagA protein into gastric epithelial AGS cells devoid of most integrin receptors on their surface, although a complete integrin-free state could not be obtained in the AGS cell background.

### KatoIII triple integrin knockout cells devoid of all αβ integrin receptors are still susceptible to CagA translocation

In order to compare our data obtained from AGS gastric epithelial cells with another independent human gastric cell line we chose the KatoIII cells for integrin gene knockout experiments. The same strategy and knockout plasmids were applied. We finally obtained a total of seven stable integrin-deficient KatoIII cell lines, all of which could be verified to be completely devoid of their corresponding cell surface integrins, as determined by flow cytometry (Fig 2F–2M). These included the single knockout cell lines (ΔITGB1, ΔITGAv, ΔITGB4), the double knockout cells (ΔITGB1B4, ΔITGAvB4, ΔITGAvB1) as well as a triple knockout cell line (ΔITGB1AvB4). The latter cell line indeed lacks all integrins we tested for by specific antibodies, as demonstrated by the absence of the αv and all individual β integrin subunits (β1 –β8) on the cell surface (see scheme Fig 1A and S7B Fig).

Next, KatoIII wild type and the corresponding knockout cell lines were analyzed by immunoblotting with the corresponding anti-integrin antibodies to confirm the complete absence of the gene product (S2B Fig). On the genetic level knockout mutations could be verified by sequencing of each gene (S1 Fig).

We then performed infection experiments to quantify CagA translocation for all seven different KatoIII integrin-knockout cell lines. Again, single or multiple integrin knockout cell lines did not show a significantly different CagA translocation efficiency as compared to wild type KatoIII cells (Fig 3B). Using a plate reader assay for adherent AGS cells, or a flow cytometry approach for KatoIII suspension cells, we next quantified and compared CagA translocation of different *Hp* P12[TEM-CagA] strains. They comprised a *hop*QI gene deficient strain (P12Δ*hopQ*[TEM-CagA]), a genetically complemented *hopQ*I knockout strain (P12Δ*hopQ*:*hopQ*[TEM-CagA]) and a strain that served as a negative control for CagA translocation (P12Δ*cagT*[TEM-CagA]). The outer membrane protein HopQ has been recently identified as a major *Hp* adhesin binding to host cell CEACAMs and was found to be a major contributing factor for CagA translocation [19, 20], whereas in AGS wild type and integrin knockout cells CagA translocation by a HopQ-deficient strain was generally reduced (Fig 3A). In contrast to AGS cells, CagA translocation by the P12Δ*hopQ*[TEM-CagA] strain into wild type KatoIII, as well as into integrin knockout KatoIII cell lines was almost completely abolished (Fig 3B). The genetically complemented strain (P12Δ*hopQ*:*hopQ*[TEM-CagA]) was restored in its ability for CagA translocation (Fig 3A and 3B).

In summary, these data support our results obtained with AGS cells. Furthermore they suggest that in AGS cells (an) other receptor(s) distinct from CEACAMs seem(s) to support the process of CagA translocation, as shown by infection assays with a P12Δ*hopQ*[TEM-CagA] strain (Fig 3A). Such (a) receptor(s) is/are apparently absent in the KatoIII cell background, where CagA translocation seems to be mostly dependent on the HopQ-CEACAM interaction (Fig 3B).

### Integrin depletion cell lines show expected surface receptor expression patterns

We next performed an integrin profiling in each integrin depletion cell line to investigate whether the depletion of individual integrins can influence the expression levels of the remaining integrins. Especially increased expression levels of remaining integrins could be a reasonable explanation for the sustained CagA translocation efficiency in the different integrin deficient cell lines. To cover all kinds of integrin combinations, also aberrant expression of non-epithelial integrin heterodimers, we analyzed integrin knockout cell lines (AGS and KatoIII background) for expression of integrin αv, β1, β2, β3, β4, β5, β6, β7 and β8 by flow cytometry using specific antibodies (S7 Fig). Among them, non-epithelial integrins β2, β3, β7 and β8 were not differently expressed by any of the mutant versus the wild type cell lines. Integrins β5 and β6 were absent only in αv knockout cells (see black arrows), probably due to the loss of their exclusive alpha integrin binding partner (S7 Fig and Fig 1A for scheme). In addition, the KatoIII β1 KO cell line showed a significant reduction in integrin αv and β5 surface localization as compared to wild type KatoIII cells (S7B Fig, black arrows). The remaining integrins expressed on the surface of each integrin-deficient cell line exhibited similar expression levels as found on wild type AGS or KatoIII cells. This reduced CEACAM expression in KatoIII knockout cells could be responsible for a slightly but not significantly reduced CagA translocation capacity of the integrin double and triple knockout cells (Fig 3B).

Elevated expression levels of remaining integrins, or aberrant expression of non-epithelial integrin heterodimers could be a reasonable explanation for the sustained CagA translocation efficiency in the integrin or CEACAM deficient cell lines. However, we can safely exclude this possibility after intensive integrin profiling experiments. Most importantly, no unexpected additional integrin subunit(s) appeared on the cell surface, even in the triple integrin knockout KatoIII cells. This indicates that these cells do not bear any integrin on their surface to be exploited for CagA translocation by *Hp*. Thus, our data unequivocally demonstrate for the first time that the apparent complete absence of any integrin on the cell surface does not have a significant effect on the capacity of *Hp* to translocate CagA into these cells.

### CEACAM1/5/6 triple knockout KatoIII cells are resistant to CagA translocation, but still able to bind *Hp*

So far our data clearly show that integrin receptors on the surface of AGS or KatoIII cells do not have a major impact for CagA translocation capacity of *Hp*, but the loss of the adhesin HopQ strongly reduced CagA translocation, especially in KatoIII cells. The question arose whether it is sufficient for CagA translocation to just mediate a physical tethering of the bacteria to the cell surface, or whether a special interplay between HopQ and CEACAMs, which may result in a very tight or close binding to the cell surface, is needed to facilitate CagA translocation?

**Fig 4:**
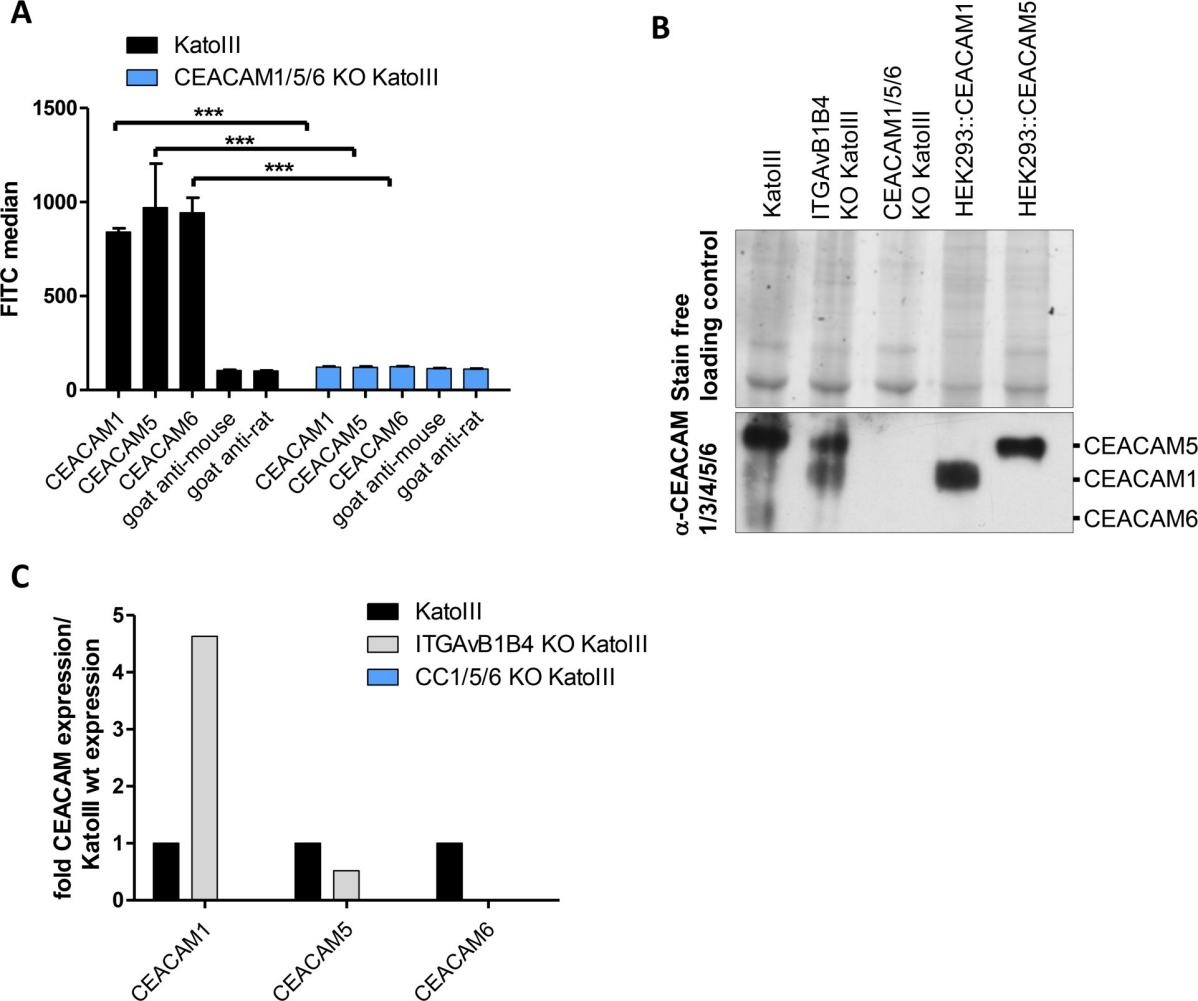
Characterization of KatoIII wild type and CEACAM- or integrin knockout cells for CEACAM expression by flow cytometry and western blotting. **A)** Wild type and CEACAM1/5/6 KO KatoIII cells were analyzed for CEACAM receptors on their surface by flow cytometry using anti-CEACAM antibodies (CEACAM1 (8G5, Genovac), CEACAM5 (26/3/13, Genovac), CEACAM6 (9A6, Genovac)). Goat anti-mouse or anti-rat were used as negative control antibodies (n = 3). For statistical analysis the Two-way ANOVA with Tukey’s HSD post-test was performed. (ns, non-significant, *** p<0.001). **B)** Immunoblot showing the production of CEACAM1, CEACAM5 and CEACAM6 by KatoIII cells and the absence of the receptors in KatoIII CEACAM1/5/6 KO cells, as indicated. The Pan α-CEACAM antibody (CEACAM1/3/4/5/6 (D14HD11, Genovac)), recognizing all three CEACAMs was used. Shown is a representative blot of an experiment that was performed three times. Lysates of HEK293::CEACAM1 and HEK293::CEACAM5 producing cells were added as controls for the correct size of the corresponding CEACAM proteins. The stainfree method was used as loading control. **C)** Densitometric quantification of CEACAM band signals in each sample in the immunoblot presented as fold CEACAM expression compared to Kato wild type cells.

To address these questions, we next generated in KatoIII cells a triple CEACAM knockout (CEACAM1/5/6 knockout) using the CRISPR/Cas system (S8 Fig) and verified the knockout status by flow cytometry, immunoblotting and sequencing (Fig 4A and 4B and S9 Fig). Attempts to combine the integrin triple knockout and the CEACAM triple knockout mutations in KatoIII were not successful, since corresponding mutant cell lines did not survive. Notably, the triple integrin knockout KatoIII cells showed a 50% reduced expression of CEACAM5 as compared to the wild type cells (Fig 4B and 4C), but a 4.5 fold increase in CEACAM1 expression, which might be explained by the fact that both surface receptors are usually found in the same lipid background and even might interact with each other [26]. This disturbance of CEACAM expression might have some influence on the slightly lower CagA translocation activity of *Hp* into the integrin double and triple knockout cells that we can always observe, although this difference is not statistically significant (Fig 3B). For CEACAM1/5/6 KO KatoIII cells no significant changes in the intrinsic integrin αv or β expression pattern could be observed (S10 Fig).

**Fig 5:**
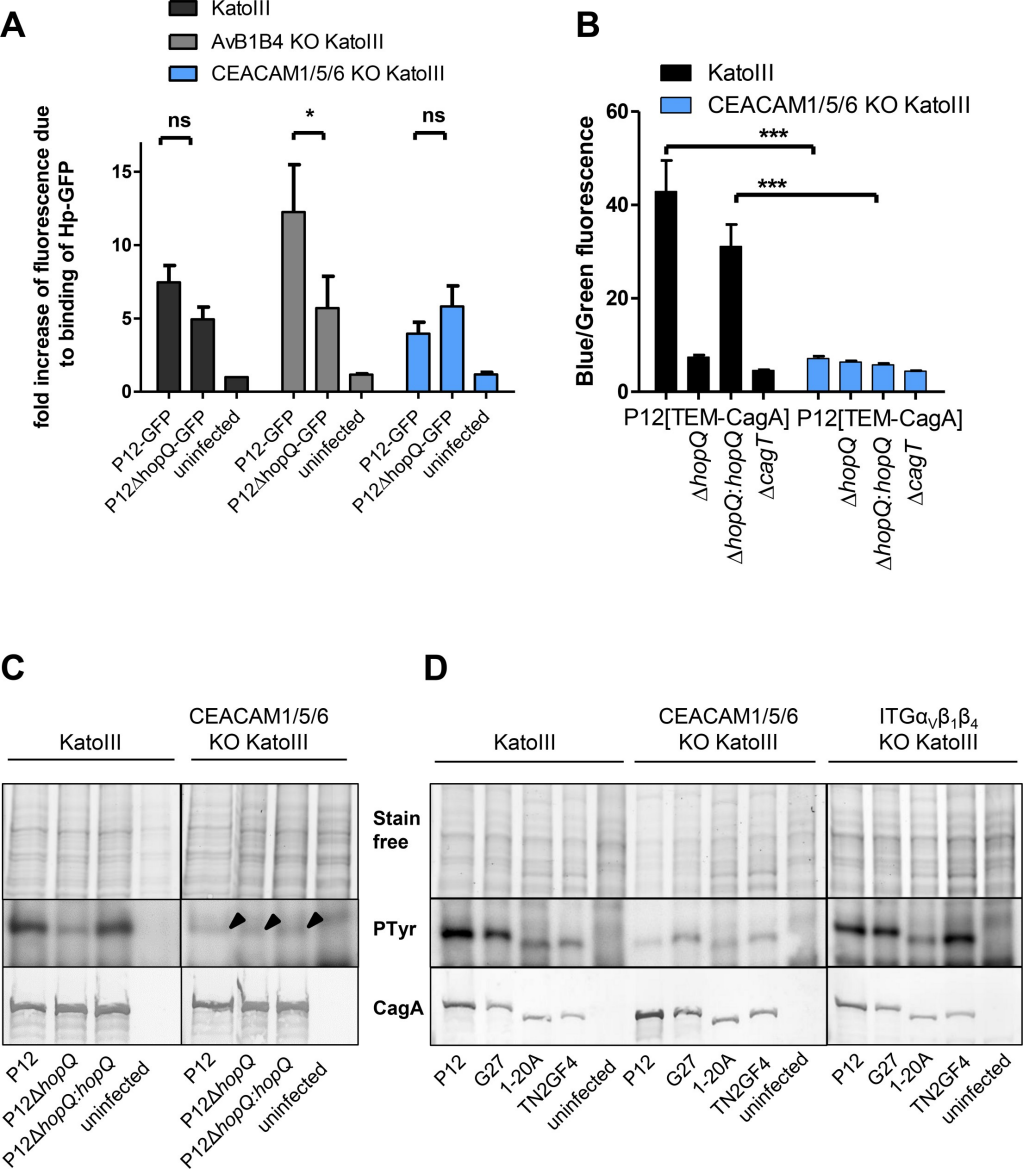
KatoIII wild type, KatoIIIΔανβ1β4 and KatoIII CEACAM1/5/6 KO cells tested for binding of P12 wt and P12Δ*hopQ* mutant strains and their CagA translocation capacity. **A**) P12-GFP and P12**Δ***hopQ*-GFP strains were used for infection of KatoIII wild type, KatoIII**Δ**ανβ1β4 and KatoIII CEACAM1/5/6 KO cells. The bacterial binding capacity of *Hp* P12-GFP and a P12Δ*hopQ*-GFP strain to the different cell lines was evaluated by flow cytometry (n = 4). The data are normalized to uninfected KatoIII cells. Statistics: Data were analyzed by Two-way ANOVA. As Post-Hoc Test a Tukey’s multiple comparison test was performed. (ns: not significant; * P < 0.05). **B)** KatoIII wild type and KatoIII CEACAM1/5/6 KO cells were infected with *Hp* P12[TEM-CagA] and corresponding mutant strains at an MOI of 60 for 2.5 h, as indicated. Ratios of blue to green fluorescence of each sample were calculated and normalized to the mean of blue to green ratio of the negative controls. All values were indicated as standard errors of the mean (±SEM) from n = 5 independent experiments. Statistics: Two-way ANOVA was performed. As Post-Hoc test mutants mean were compared by a Bonferroni test (ns: not significant *** P < 0.001). **C)** KatoIII cells or the CEACAM1/5/6 KO cell line were infected with strain P12, P12Δ*hopQ* or P12Δ*hopQ*:*hopQ*, for 2.5 hours with an MOI of 60. Translocation of CagA was determined by detecting tyrosine-phosphorylated CagA with the antibody PY99. Arrowheads indicate the position of the weak tyrosine-phosphorylated (PTyr) CagA band. **D)** KatoIII cells or the triple integrin-depletion KatoIII cell line were infected with strain P12, G27, 1-20A or TN2GF4 for 2.5 h with an MOI of 60. Translocation of CagA was determined by detecting tyrosine-phosphorylated CagA with the antibody PY99.

We then infected the CEACAM triple knockout KatoIII cells with the P12[TEM-CagA] strain to quantify CagA translocation. As expected from the results with the P12Δ*hopQ*[TEM-CagA] strain, the CEACAM triple knockout showed a nearly complete loss of CagA translocation, comparable to and in support of the P12Δ*hopQ*[TEM-CagA] strain results (Figs 3B and 5B). Similar results were obtained in the CagA tyrosine phosphorylation assay for strain P12 and other *Hp* lab strains (Fig 5C and 5D). Thus, in KatoIII cells the HopQ-CEACAM interaction seems to be the major driver/mediator for CagA translocation.

### Quantitative differences in adherence and minor changes in the general binding pattern of *Hp* to KatoIII wild type, integrin- or CEACAM-deficient cells

Next, an important question was how much of the total adhesion of the bacteria can be attributed to the HopQ-CEACAM interaction and is binding per se, independent of the type of host cell receptor, sufficient to allow CagA translocation. Interestingly, the binding capacity of a P12-GFP strain to KatoIII wild type versus the CEACAM triple knockout KatoIII cells was reduced to a level of about 75% (Fig 5A), whereas the CagA translocation was nearly completely abolished under these circumstances (Fig 5B). These data suggest that binding per se is not sufficient for *Hp* to induce CagA injection. It seems that the HopQ-CEACAM interaction mediates a(n) additional signal(s) to initiate CagA injection.

**Fig 6:**
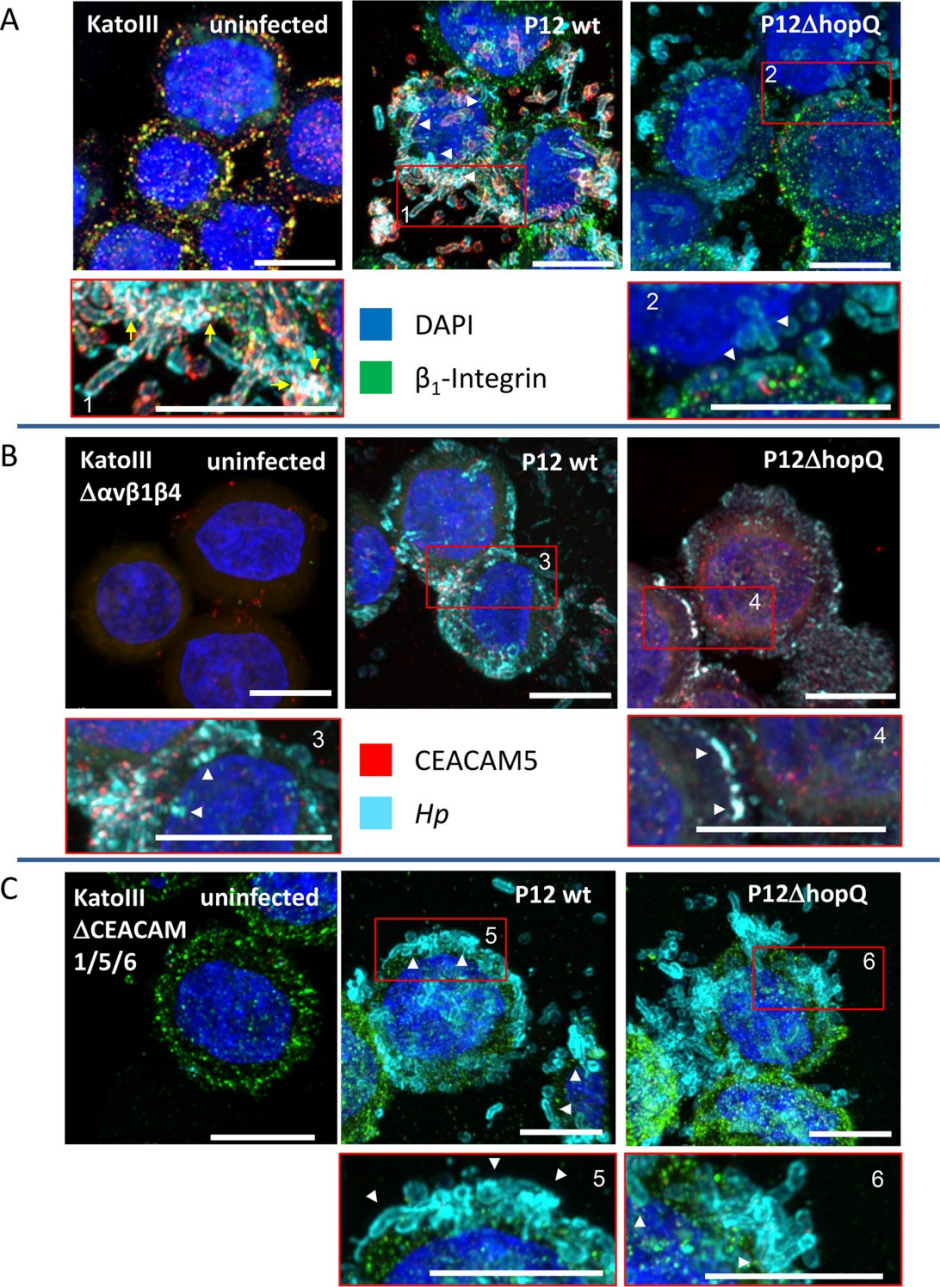
Confocal laser scanning microscopy (CLSM) studies of the epithelial cell line KatoIII and integrin and CEACAM knockout KatoIII cells infected with *Hp* P12 wild type and mutant strains. Confluent monolayers of KatoIII (**A**), KatoIIIΔαvβ1β4 (**B**) and or KatoIIIΔCEACAM1/5/6 (**C**) cells were infected with *Hp* P12 wt, P12Δ*hopQ* or P12Δ*hopQ*::*hopQI*. The cells were analyzed by CLSM (63 x objective) by a Zeiss LSM 880 with Airyscan. The top row shows an overview, the lower row a magnification of characteristic observation fields specified by a red window. Several planes are combined to obtain a Z-stack (bottom to top), showing bacterial binding and co-localization of β1 integrin or CEACAM5 and *Hp*. White arrowheads point to the close association of *Hp* with the gastric epithelial cell surface, yellow arrows point to co-localization events between *Hp* and CEACAM. Scale bars represent 10 μm. At least 3 micrographs of independent cell culture samples were taken, one representative area is shown. Colors for co-localizations: β1-Integrin/CEACAM5 (green/red): yellow; CEACAM5/ *Hp* (red/petrol-light blue): white; β1-Integrin/CEACAM5/*Hp* (green/red, petrol-light blue): white.

To further study potential changes in the interaction of *Hp* with cells lacking all surface integrin receptors, or the relevant CEACAM receptors, we performed confocal microscopy studies using KatoIII wild type cells, KatoIIIΔαvβ1β4 and KatoIIIΔCEACAM1/5/6 cell lines infected with P12 wild type or P12Δ*hopQ* strains (Fig 6A–6C). We typically find a reduced number of *Hp* binding to KatoIIIΔαvβ1β4 and KatoIIIΔCEACAM1/5/6 cell lines as compared to KatoIII wild type cells, and the binding pattern of *Hp* to integrin-deficient cells appears to be different. Interestingly, KatoIIIΔCEACAM1/5/6 cell lines produce large amounts of β1 integrin (Fig 6C), and *Hp* is found closely attached to β1 integrin, although under these conditions very little CagA translocation was found (Fig 5B). Thus, we see for each cell line an intimate interaction of the bacteria with the host cells, independent of the capacity for CagA translocation of the strain (Fig 6A–6C, white arrowheads). Triple integrin knockout cells show a high number of adherent bacteria (Fig 5A) but a lower expression of CEACAM5 (Fig 4B and 4C). This is also visible by a lack of CEACAM5 recruitment to the bacterial surface in the triple integrin knockout cells, which is in stark contrast to the CEACAM5 receptor recruitment seen in *Hp-*infected KatoIII wild type cells (Fig 6A, versus B and C; yellow arrows). Notably, CagA translocation into these cells is not significantly reduced as compared to the KatoIII wild type cells (Fig 3B and 3C).

## Discussion

It is well established that the *cag*PAI-encoded T4SS is a major *Hp* virulence determinant, the function of which has been implicated in severity of disease and increased risk of gastric cancer [27]. A major role of the *cag*-T4SS is the translocation of the CagA protein into various types of host cells, where CagA interferes in a phosphorylation-dependent and phosphorylation-independent manner with signaling events to manipulate fundamental processes in the gastric epithelium [28]. Major outcomes include the suppression of innate defense mechanisms [29], changes in cell polarity and migration [30, 31], and putatively oncogenic events [32, 33]. The involvement of a host cell integrin heterodimer (α5β1 or any other β integrin heterodimer) acting as receptor for the *Hp* T4SS, especially for the pilus-associated RGD containing CagL protein, was considered as a major requirement for CagA translocation [13, 14] [34, 35]. Several labs have provided data showing the interaction of integrin α5β1 or other αβ integrin heterodimers with different components of the *cag*-T4SS, especially CagL [13, 34–41], but also CagA [16], CagI [42] and CagY [14, 42]. Several previous studies suggested that integrins are required for CagA translocation. The major evidence for a functional role of β1 integrins as receptor for the *cag*-T4SS and translocation of CagA was coming from studies using β1 integrin-deficient murine fibroblast (GD25) or epithelial (GE11) cell lines, which did not support CagA translocation, whereas the corresponding β1 integrin complemented versions resulted in CagA translocation and phosphorylation [13, 14]. We report now that integrin heterodimers are not required for *Hp* to translocate its CagA into gastric epithelial cell lines *in vitro*.

We were interested in a better understanding of the role and the contribution of the recently identified CEACAM receptors versus the integrin receptors for CagA translocation. Therefore we generated a set of knockout cell lines and measured their ability for CagA translocation. The β-lactamase reporter system determines *Hp* CagA translocation into host cells in a very sensitive, reproducible and quantitative way [25]. Most important, it measures the translocation of CagA directly, rather than its tyrosine phosphorylation. The tyrosine phosphorylation depends on the activity of host cell kinases c-Src or c-Abl, which might be affected in their activity by manipulations of the cells, such as different growth conditions, buffer treatments or procedures like the gene knockout technology.

Taking advantage of the CRISPR/Cas technology we started to knock out important integrins in an additive fashion to generate single (β1), double (β1β4, αvβ4, β1αv,) AGS and KatoIII cells and finally a triple integrin gene knockout cell (αvβ1β4) in the KatoIII background. All CRISPR/Cas constructs and targeted cell lines for the generation of integrin-depletion AGS and KatoIII cell lines are summarized in S2 Table. AGS cells are adherent cells, which did not survive as triple knockout without any integrin on the surface. In AGS cells growing as attached cells on solid surfaces this phenomenon might be due to the induction of anoikis, a tissue architecture surveillance mechanism, which can be induced by the absence of integrin-ECM ligation to assure that dissociated and displaced cells are effectively eliminated, in order to prevent dysplastic growth [43, 44]. KatoIII cells, which grow in a semi-adherent manner, were resistant to anoikis and allowed the generation of a triple integrin knockout. The triple integrin knockout KatoIII cell line, which is devoid of any integrin receptor on the surface, was still competent for CagA translocation. From these results we have to conclude that neither the direct interaction of components of the *cag*-T4SS with integrins, nor any integrin-mediated signaling event is necessary for CagA translocation.

The data presented above seem to be in opposition to earlier publications [13, 14], which demonstrated that CagA translocation was possible in murine β1 integrin expressing GE11 or GD25 cells, but not in the corresponding integrin knockout cells. However, neither the murine GE11 and GD25, nor the chinese hamster ovary (CHO) cell line, also used for such experiments, contain human CEACAMs for binding of the *Hp* adhesin HopQ. This might at least explain why in our earlier experiments only very low (background) levels of CagA translocation could be observed in the β1 integrin-complemented versions of these cells [14]. From our new perspective, the earlier integrin complementation data can be interpreted that the interaction of the *cag*-T4SS to integrins in cells without human CEACAM receptors has only a small supportive effect for CagA translocation. However, the integrin knockout data presented in this study clearly show that integrins are not necessary for CagA translocation in AGS or KatoIII gastric cell lines.

Using hamster (CHO) cell lines devoid of human beta integrins, but genetically complemented with integrin genes encoding fully functional or partial integrins (CHO K1, CHOβ1TR) we demonstrated that the extracellular part of β1 integrin was supportive for CagA translocation, but the cytoplasmic tail of β1 integrin was not necessary [14]. We also reported that neither the RGD motif in CagL for binding the β1 integrin heterodimer, nor the function of the integrin linked kinase (ILK) were essential for CagA translocation. From that we concluded that no integrin-mediated signaling is involved in this process [14]. Interestingly, also knockdown experiments of integrin α5β1 and ILK showed that both were dispensable for NF-κB activation during *Hp* infection, but the bacterial adhesin HopQ promoted canonical NF-κB activation in AGS and NCI-N87 cells [45]. Combined these data suggest that integrin-mediated signaling is neither needed for CagA translocation nor NFκB activation.

Earlier data also showed that a recombinant protein of CagA covering the binding site of β1 integrin can interfere with CagA translocation and phosphorylation [16]. A similar effect on CagA phosphorylation was seen with the β1 integrin-specific antibody 9EG7 [14]. These data were interpreted as direct effects of β1 integrin on CagA translocation, suggesting that β1 integrin is essential. In the context of our results in this study we would explain these data as more indirect effects, e.g. by steric hindrance exerted by the binding of the recombinant protein, or the 9EG7 antibody, on the function of other receptors, such as CEACAMs, which usually reside in the same lipid domains as integrins [26].

With these novel results the question arises why components of the *cag*-T4SS bind specifically and in some cases with high affinity (CagA, K_D_ values in low nanomolar range) to α5β1 integrin heterodimers [14, 42] although this binding apparently has only a very minor, functional relevance for CagA translocation? The binding of the *cag*-T4SS components to the extracellular domains of β1 integrin heterodimers may allow, by tethering of the T4SS to the host cell, a low level CagA translocation, but for a full CagA translocation, the HopQ adhesin–CEACAM binding is necessary. Furthermore, we cannot exclude that in an *in vivo* situation, when *Hp* interacts with primary gastric cells in tissue, integrin signalling via CagL might play a role. CagA translocation might happen independent from integrin interaction, as our *in vitro* data suggest, but the activation of Src kinase might be necessary in primary, untransformed cells, but dispensable in transformed cell lines, in which these kinases often are constitutively active.

Our group as well as other labs have shown that certain CEACAMs act as receptors for *Hp* and support CagA translocation when they are reconstituted in a cell line deficient of CEACAM expression (e.g. HEK293, CHO) [18–20]. The genetic complementation of CEACAM-negative cells, such as CHO or HEK293 cells, showed a drastic effect on CagA translocation [18–20]. However, in contrast to the integrin knockouts, a complete genetic knockout of CEACAM1/5/6 in an epithelial cell line (KatoIII) more or less completely abrogated CagA translocation. This clearly suggests that CEACAM receptors are essential for CagA translocation in certain cell types.

Besides CEACAMs, other surface receptors seem to exist, which can support CagA translocation. Thus, in AGS cells we see a reduction in CagA translocation to approximately 50% when a HopQ-deficient versus a wild type *Hp* strain is used for infection (Fig 3A) [19], which is in contrast to KatoIII cells suggesting that in AGS gastric epithelial cells an additional, so far unknown receptor might be expressed, which is probably targeted by another *Hp* adhesin to support CagA translocation. This receptor might be absent in other cell lines, such as KatoIII cells. Earlier work described a small BabA-Leb mediated but *cag*-T4SS-dependent effect on the production of proinflammatory cytokine mRNA expression (IL-8, CCL5) and a very minor effect on CagA translocation of *Hp*. This Leb dependent augmentation of *cag*PAI T4SS functions, which was seen in Leb-negative non-human and non-gastric CHO or Madin-Darby canine kidney cells (MDCK) transfected with several glycosyltransferase genes [46], was independent of CEACAMs, since these cells do not produce CEACAMs recognized by HopQ.

Besides the interaction of the bacteria with protein- or oligosaccharide cell surface receptors, the translocated effector protein CagA can bind phospholipids via a K-Xn-R-X-R motif, an amino acid sequence motif conserved among various pleckstrin homology (PH) domains directly involved in the interaction with acidic phospholipids, such as phosphatidylinositol (PI) and/or phosphatidylserine (PS) [47]. Murata-Kamiya and coworkers [48] reported that physical interaction of *Hp* CagA with host membrane PS, which is aberrantly externalized at the site of bacterial attachment by *Hp*, plays a key role in the delivery and intracellular localization of CagA. How the exploitation of CEACAM receptors by the adhesin HopQ, the functional buildup of the *cag*-T4SS secretion apparatus and the CagA binding to PS are coordinated and function to accomplish the internalization of CagA is still not well understood and the aim of intensive future research.

## Materials and methods

### Bacterial strains and culture conditions

For TEM-1 reporter assays *Hp* wild type strain P12 [49] and defined P12 knockout mutants were used. To verify CagA translocation results into integrin-deficient AGS or integrin- or CEACAM-deficient KatoIII cells by tyrosine phosphorylation also other *Hp* strains were applied, such as 1-20A, TN2GF4 or G27 [50]. These strains harbor a functional *cag* pathogenicity island (*cag*PAI) in their genome, encoding the Cag T4SS [4]. *Hp* strains and mutants used in this study are listed in S3 Table.

*Hp* strains were grown on GC agar plates (Oxoid) supplemented with vitamin mix (1%) and horse serum (8%) (serum plates) and cultured for 16–60 h in a microaerobic atmosphere (85% N_2_, 10% CO_2_, 5% O_2_) at 37°C. *Escherichia coli* strains Top10 and DH5alpha were grown on Luria–Bertani (LB) agar plates or in LB liquid medium [51] supplemented with antibiotics, as appropriate. Cell lines were cultivated under standard conditions [15] in 75 cm^2^ tissue culture flasks (BD Falcon) and subcultivated every 2–3 days in 6-well, 48- well (tissue culture treated, Costar, Corning Inc.) or 96-well microtiter plates (black, transparent bottom, tissue culture treated, 4titude). Plasmids were introduced into *Hp* strains by transformation as described previously [52]. *Hp* transformants were selected on serum plates containing 6 mg l^-1^ chloramphenicol, 8 mg l^-1^ kanamycin, 10mg l^-1^ erythromycin or 250 mg l^-1^ streptomycin, as appropriate.

### Cultivation and maintenance of cells

AGS cells (CRL-1739) and Kato III cells (HTB-103) were obtained from ATCC. Cells were generally cultured in RPMI 1640 or DMEM supplemented with 10% (vol/vol) fetal calves serum (FCS) at 37°C and 5% CO_2_. For passaging of the cells, the medium was removed and the cells were gently washed once with Dulbecco´s PBS (DPBS, Life Technologies). To detach cells, 2 ml Trypsin-EDTA was added to a 75 cm^2^ flask for 3–5min incubation at 37°C. When detachment was observed under microscope, 8 ml of the pre-warmed RPMI1640 medium was added to neutralize the trypsin. After being gently pipetted up and down, cells were dissociated and were then reseeded into new flasks. Passages taken place in every 2–3 days with a split ratio of 1:5 or 1:8. Cells were discarded when the passage number reached 80.

### Transfection of adherent/semi-adherent cells by Lipofection

Generally, one day before transfection, 0.5 x 10^5^ to 2 x 10^5^ cells were plated in 500 μl of growth medium without antibiotics in a 24-well plate so that they would be 90–95% confluent at the time of transfection. For each transfection sample, 500 μg DNA and 2 μl lipofectamine 2000 was prepared according to the manufacturer´s instructions (Lipofectamine 2000, Invitrogen). After transfection the cells were incubated at 37°C in a CO_2_ incubator for 24–48 hours until they were ready to test for transgene expression.

### Detection of proteins on the cell surface by flow cytometry

Cells were counted and added to a round-bottom 96-well plate with 2x10^5^ cells per well. After centrifugation (300 g at 4°C) primary antibodies were added to each well following the recommended concentration from the manufacturer. Dilutions of antibody, if necessary, were made in FACS buffer. Cells and antibodies were incubated at 4°C for 1 hour in the dark. Primary antibody stained cells were washed 3 times and resuspended in 200 μl to 1 ml of ice-cold FACS buffer. Subsequently, fluorochrome-labeled secondary antibodies were diluted in FACS buffer at the optimal concentration (according to the manufacturer’s instructions) and were added to each well, followed by 1 h incubation at 4°C in the dark and 3 times washing as described above. Cells were analyzed by flow cytometer right after washing or kept in the dark on ice until the scheduled time for analysis. The following antibodies were used:

ITGA2 (P1E6, chemicon), ITGA3 (P1B5, chemicon), ITGAv (P2W7, Santa Cruz), ITGB1 (AIIB2, EMD Millipore), ITGB2 (MEM-48, antibodies-online GmbH) ITGB3 (VI-PL2, antibodies-online GmbH), ITGB4 (439-9B, antibodies-online GmbH), ITGB5 (AST-3T, antibodies-online GmbH), ITGB6 (437211, antibodies-online GmbH), ITGB7 (FIB504, antibodies-online GmbH), ITGB8 (416922, antibodies-online GmbH), CEACAM1 (8G5, Genovac), (CEACAM5 (26/3/13, Genovac), CEACAM6 (9A6, Genovac).

### Generation of integrin- and CEACAM-depletion cell lines

#### Design of short guide RNA

Design of short guide RNA (sgRNA) for gene targeting by the CRISPR/Cas9 system was accomplished using an online design tool (http://tools.genome-engineering.org) developed by Feng Zhang’s lab in Broad Institute of Massachusetts Institute of Technology and Harvard. Firstly, a 23 to 250 bp genome fragment from the target region was fed into the design tool. After computational analysis, suitable targets were identified and listed by ranking and scores, according to the prediction of their off-target potential. Usually, the input region of interest should be selected from promoter region or early exons of the target gene. Designed sgRNAs were ordered as oligos commercially. To obtain functional sgRNAs, more than one pair of sgRNAs were designed for integrin beta1 target gene and their efficiencies were tested in the intended cell line (see S1 Table).

In order to acquire sgRNA expressing constructs, top and bottom oligos were annealed before cloning (e.g. Fig 1D). For each reaction, 100 μM top oligo, 100 μM bottom oligo and 2 μl 10 ×T4 ligation buffer were mixed thoroughly. Water was added to each mixture to a total volume of 20 μl. The reaction was heated (95°C for 5 min) followed by slow cooling (4–8°C) to allow the temperature ramping down naturally. The CRISPR vector used in this study was the Cas9 nickase vector (pSpCas9n(BB)-2A-Puro, PX462). The vector harbors the conserved region of the remained sgRNA scaffold, *S*. *pyogenes* Cas9 nickase, along with the 2A-Puro for transfection selection. After restriction digestion of the vector (Bpil) and ligation of annealed oligos, Stbl3 competent cells (Invitrogen) were transformed by heat shock. After the outgrowth, 100 μl of each transformation was plated on ampicillin LB plates. Colony growth was inspected and two to three colonies from each transformation were restreaked for sequence verification from the U6 promoter on the vector using U6-Fwd primer (see S1 Table).

#### Functional validation of sgRNAs

Functional validation of sgRNAs was completed by transfection of sgRNA expressing constructs into target cell lines, followed by verification of genome modification from the transfected population. In this study, host cell surface expressing integrins were the targets of CRISPR-Cas9 mediated gene knockout. Therefore, verification of genome modification was accomplished by flow cytometry surface integrin detection. When the transfected population showed two distinct populations with different phenotypes, in this case, one of the populations with a specific integrin expression and the other without, sgRNA(s) were considered valid and efficient. In order to clarify whether a newly designed sgRNA was working as expected, which means that it targets the expected integrin gene, we checked the transfected cell population early after transfection by flow cytometry. In that state the population was still heterogeneous, but when cells with a specific integrin and a certain percentage of cells without the corresponding integrin expression were detected, we considered the sgRNA construct as functional. After that we isolated a homogeneous knockout population from such a pool by FACS sorting for cells lacking the integrin.

#### FACS sorting for desired population

Generation of stable cell lines started from FACS sorting for integrin negative populations from transfected cells. Transfected cells went through a defined selection procedure in order to obtain knockout cell lines. Since CRISPR constructs contain the puromycin resistance gene, the transfected population was treated with puromycin to kill non-transfected cells. The surviving cells were stained with integrin β1 antibody for negative selection by FACS sorting. Finally, serial dilutions of the sorted negative populations resulted in stable cell lines, which could be verified as completely integrin β1-deficient by flow cytometry analysis. After sorting, most of the cells with an undesired phenotype were removed, in a way to markedly simplify the time- and labor-consuming selection works. Each transfected population was stained with specific anti-integrin or anti-CEACAM antibodies for sorting. After sorting, cells were cultured in the presence of penicillin and streptomycin for one or two weeks until they reached the number of 1 × 10^6^ for long term storage by freezing in liquid nitrogen.

#### Generation of integrin- and CEACAM-depletion cell lines

Stable cell lines which arose from a single, two or three knockout cells, were obtained by performing serial dilutions from the sorted integrin- or CEACAM-negative population. Sorted cells were detached and dissociated by pipetting up and down carefully to prevent clumping. Afterwards, the cell number was determined by counting with a hemocytometer. In order to dilute the cells in a final concentration of statistically 1.5 cell per well in a 96-well plate, 150 cells were resuspended in 22 ml complete medium and 200 μl diluted cells were added to each well. At least two 96-well plates were plated for each sorted population. One to two weeks later, colonies in each well were inspected with the microscope, and those wells with more than three colonies were marked off. Plates were returned to the incubator to allow them to grow for another 1 to 2 weeks. The wells with one to three clones were marked and expanded to 48- well plates, then 24-well plates, then 6-well plates and finally 25 cm^2^ flasks for examination and freezing.

### Quantification of TEM-1 CagA translocation and plate-reader detection

This procedure is used for adherent cells, such as AGS cells. One day before infection, adherent cells were detached and 2.5 × 10^4^ cells were seeded in each well in a 96-well plate with black wall and transparent bottom with low fluorescence background (4ortitude). The confluence of the cells was 80% to 90% on the day of infection. Before infection, *Hp* strains with fusion protein of beta-lactamase TEM-1 and CagA were collected as described before. Ideally, bacteria were resuspended and pre-incubated in sterile PBS containing 10% FCS at 37°C, 10% CO_2_ for 1.5 h. Subsequently, cells were infected by bacteria with an MOI of 60 for 2.5 h at 37°C, 5% CO_2_ as described above. Infections were stopped by placing the plates on ice and all the supernatants were removed. Prepared substrates mix is loaded immediately on the cell surface, followed by incubation at room temperature for 120 min in the dark. Plate reader filters are set to allow excitation of wavelength around 410nm, and detection of blue emission around 450nm and green emission around 520nm. Afterwards acquired data was normalized and analyzed following manufacturer’s instruction to obtain the blue to green fluorescence ratio.

### Quantification of TEM-1 CagA translocation with flow-cytometry detection

For suspension and semi-adherent cell lines, CagA translocation was detected by flow cytometry. The method of CagA translocation assay with flow-cytometry detection is very similar to the plate-reader detection except following procedures. Firstly, semi-adherent cells were detached after infection with room-temperature trypsin-EDTA before incubation with CCF4-AM fluorescence substrate mix. Secondly, incubation of cells with CCF4-AM mix were implemented at 27°C with constant shaking condition to allow even loading of cells with substrate and avoid cell sedimentation. Finally, cells were washed at least 2 times with PBS by centrifugation at 200–300 × g for 5 mins after incubation with CCF4-AM substrate. Cells were then analyzed by flow cytometry for Pacific Blue fluorescence and AmCyan green fluorescence.

### Antibodies, infection of cell cultures and immunoblotting

Rabbit polyclonal antisera AK268 and AK257, directed against the CagA N-terminal and the CagA C-terminal region, respectively, have been described previously [5, 53]. The mouse monoclonal antibody against TEM-1 β-lactamase was obtained from Abcam (ab12251). For immunoblotting the following antibodies were used: ITGAv, (EPR16800, abcam), ITGB1 (LM534, EMD Millipore), ITGB4, (439-9B, abcam), CEACAM1/3/4/5/6 (D14HD11, Genovac).

Standard infections of AGS and KatoIII cells with *Hp* strains and subsequent preparations for phosphotyrosine immunoblotting were performed as described previously [23]. Briefly, cells were plated one day before infection in 6-well plates. On the day of infection, cells were infected using an MOI of 60 for 2.5 h at 37°C and 5% CO_2_. After washing with PBS, cells were collected by cell scrapers in the presence of 1 ml PBS* (PBS containing 1 mM Na_3_V0_4_, 1 mM PMSF, 10 μg/ml leupeptin and 10 μg/ml pepstatin). Cells with adherent bacteria were collected by centrifugation and resuspended in SDS-PAGE sample solution.

### Sodium dodecyl sulfate–polyacrylamide gel electrophoresis (SDS-PAGE) and Western blotting

SDS PAGE and western blotting was performed as described previously [13]. For the development of immunoblots, polyvinylidene difluoride (PVDF) filters were blocked with 5% non-fat milk powder in TBS (50 mM Tris–HCl, pH 7.5, 150 mM NaCl), 0.1% (v/v) Tween 20 (TBS-T), and incubated with the respective antisera at a dilution of 1:1.000–1:15.000 in TBS-T with 1% non-fat milk powder. Horseradish peroxidase-conjugated anti-rabbit IgG antiserum was used to visualize bound antibody. Standard infections of AGS and KatoIII cells with *Hp* strains and subsequent preparations for phosphotyrosine immunoblotting were performed as described previously [5]. Tyrosine-phosphorylated proteins were analyzed by immunoblotting with the phosphotyrosine antibody PY99 (Santa Cruz Biotechnologies).

### StainFree staining

Single gel systems [54] were adapted for Stain-Free detection as described in protocol depository Protocols.io under dx.doi.org/10.17504/protocols.io.gipbudn.

### Quantification of signals in Western Blots

Western Blot data were quantified by densitometry using ImageJ. Band intensities of strain-free gel were normalized to the band intensity of KatoIII lane. CEACAM1, 5 and 6 expression was measured as area percent of the respective lane and normalized to the CEACAM1, 5 and 6 expression of KatoIII cells. Comparability between cell lines was achieved by standardizing each normalized CEACAM expression to the normalized loading controls.

### Microscopy

One day prior to experiments cells were seeded at 5 x 10^4^ cells in a 24-well plate equipped with uncoated cover slides and grown overnight at 37°C and 5% CO_2_. Cells were infected with *Hp* wild type or isogenic mutant strains with an MOI of 10 for 3h at 37°C and 5% CO_2_. For immunostaining cells were fixed with 4% PFA for 10 min at room temperature. Cells were washed twice with Dulbecco´s PBS (DPBS, Life Technologies) and blocked overnight with 2% FCS in PBS at 4°C. Fixed cells were incubated with mouse anti-CEACAM5 (26/3/13, Genovac, 1:300), rabbit anti-*Hp* (AK175, 1:400) and rat anti-integrin beta1 (AIIB2, Millipore, 1:200) for 1h at room temperature. After washing secondary antibodies were applied (goat anti-rat Alexa488, goat anti-mouse Alexa555 and goat anti-rabbit Alexa647 all from Invitrogen, 1:1000) and incubated for 1h at room temperature in the dark. Cell nuclei were stained with DAPI (5μg/ml) for 10 min. Samples were mounted on the cover slip with Fluorescent Mounting Medium (DAKO). A cytospin3 (Shandon) was used to centrifuge suspension cells onto glass slides. Micrographs were taken with a confocal laser scanning microscope (LSM880, Zeiss) with Airyscan Module using a 63x oil immersion objective.

### Statistical analysis

Statistical analysis was performed with GraphPad Prism 7.2. Data were analyzed with One-way or Two-way analysis of variance (ANOVA), as further specified in the legends of the corresponding figures. The significance level was set to 0.05. If overall ANOVA tests were significant, a post hoc test (Tukey’s HSD test or Bonferroni test) was performed. Details for each experiment are described in the figure legends.

## Supporting information

S1 Fig Verification of targeted deletions within integrin genes of AGS and KatoIII cells by gene amplification and DNA sequencing. The top line shows the corresponding sequence of human integrin β1 **A**), the integrin αv **B**) or the β4 gene **C**) showing the Guide A and Guide B sequences (blue, underlined), the PAM sequence and putative cleavage sites of Cas9 nickase. (red arrowheads). The deleted areas as identified by sequencing of corresponding PCR fragments are indicated by a dashed line.
(TIF)

S2 Fig Verification of the loss of integrin and CEACAM protein production by immunoblotting. Lysates of AGS wild type and integrin knockout cell lines (**A) and** KatoIII wild type and integrin- or CEACAM1/5/6 knockout cell lines **(B)** were analyzed by immunoblotting using specific antibodies against human integrins as indicated. Loading controls are presented by the stain-free method on top using corresponding cell lysates.
(TIF)

S3 Fig Strategy for targeted deletion of integrin αv gene in exon 4. *Streptococcus pyogenes* Cas9 nickase binding sites (20 bp, highlighted in blue) are immediately followed by the 5’-NGG PAM (protospacer adjacent motif). The short guide RNA (sgRNA) pairs are located on both strands of the target DNA with a 25 bp gap. Cloning scheme of the CRISPR plasmids (see Materials and methods for details).
(TIF)

S4 Fig Strategy for targeted deletion of integrin β4 gene in exon 6. *Streptococcus pyogenes* Cas9 nickase binding sites (20 bp, highlighted in blue) are immediately followed by the 5’-NGG PAM (protospacer adjacent motif). The short guide RNA (sgRNA) pairs are located on both strands of the target DNA with a 25 bp gap. Cloning scheme of the CRISPR plasmids (see Materials and methods for details).
(TIF)

S5 Fig Characterization of AGS wild type and integrin knockout cell lines for their ability to induce the hummingbird phenotype. **(A)** AGS wild type, AGS αvβ4 or AGS β1β4 cells were infected with P12 wt, P12Δ*hopQ*, or a complemented P12Δ*hopQ*/*hopQ Hp* strain re-expressing wt *hopQ* gene for 4 h. As compared to non-infected controls, AGS wild type and AGS knockout mutant cells show an elongated and spindle-shaped (hummingbird) phenotype. Bar, 50 μm.
(TIF)

S6 Fig Determination of IL-8 induction in AGS integrin-depletion cell lines. The induction of IL-8 was determined after infection of AGS wild type or integrin knockout cell lines for 4 h with P12 wt, P12Δ*hopQ*, P12Δ*hopQ*/*hopQ* or other *Hp* lab strains. Statistics: n = 4, one way Anova, ***, p<0.001. Values are means +/- SEM.
(TIF)

S7 Fig Integrin profiling in different integrin-depletion cell lines. Wild type cell lines and integrin-depletion cell lines were stained with antibodies specific to ITGAv, ITGB1, ITGB2, ITGB3, ITGB4, ITGB5, ITGB6, ITGB7 and ITGB8, and were subsequently monitored by flow cytometry in the FITC-A channel. FITC median were obtained and analyzed with the Flowjo software. All values were indicated as standard errors of the mean (+SEM) from three independent experiments. The significance of differences was analyzed using One way ANOVA. **A)** Integrin profiling in integrin-depletion AGS cell lines (n = 3). **B)** Integrin profiling in integrin-depletion KatoIII cell lines (n = 3). Integrin β subunits, which were strongly reduced, or completely absent in certain knockout cell lines, are marked with black arrows.
(TIF)

S8 Fig Strategy for a targeted deletion within exon 2 of the CEACAM1 gene in KatoIII cells. *Streptococcus pyogenes* Cas9 nickase binding sites (20 bp, highlighted in blue) are immediately followed by the 5’-NGG PAM (protospacer adjacent motif). The short guide RNA (sgRNA) pairs are located on both strands of the target DNA with a 25 bp gap. Cloning scheme of the CRISPR plasmids (see Materials and methods for details).
(TIF)

S9 Fig Verification of targeted deletions within the CEACAM1, CEACAM5 and CEACAM6 genes of KatoIII cells by gene amplification and DNA sequencing. The top line shows the corresponding sequence of human CEACAM1 **(A)**, CEACAM5 **(B)** and CEACAM6 gene **(C)** with the Guide A and Guide B sequences (blue, underlined), the PAM sequence and putative cleavage sites of Cas9 nickase. (red arrowheads). The deleted areas as identified by sequencing of corresponding PCR fragments are indicated by a dashed line.
(TIF)

S10 Fig Integrin profiling in KatoIII wild type and KatoIII cells lacking CEACAM1, CEACAM5 and CEACAM6 (CEACAM1/5/6 KO) cells. KatoIII cells and integrin-depletion cell lines were stained with antibodies specific to ITGB1, ITGB2, ITGB3, ITGB4, ITGB5, ITGB6, ITGB7 and ITGB8, and ITGAv and were subsequently monitored by flow cytometry in the FITC-A channel. FITC median were obtained and analyzed with the Flowjo software. All values were indicated as standard errors of the mean (+SEM) from three independent experiments. The significance of differences was analyzed One way ANOVA with Tukey’s HSD post-test.
(TIF)

S11 Fig Quantitative evaluation of CagA translocation into wild type integrin-knockout AGS or KatoIII cell lines by the TEM-1 β-lactamase reporter assay. **A)** Raw data of KatoIII cells and derivatives thereof measured by flow cytometry, as shown in Fig 3A. **B)** Raw data of KatoIII cells and derivatives thereof measured by flow cytometry, as shown in Fig 3B.
(TIF)

S1 Table Sequences of paired sgRNAs designed for targeting ITGB1, ITGAv and ITGB4 genes. (PDF)

S2 Table CRISPR constructs and targeted cell lines for the generation of integrin-depletion AGS and KatoIII cell lines. (PDF)

S3 Table Bacterial strains used in this study. (PDF)
