# Supplementary figures and images for: Integrin but not CEACAM receptors are dispensable for Helicobacter pylori CagA translocation
Source: PLoS Pathog. 2018 Oct 26;14(10):e1007359. doi: 10.1371/journal.ppat.1007359 (PMC6231679; doi:10.1371/journal.ppat.1007359)

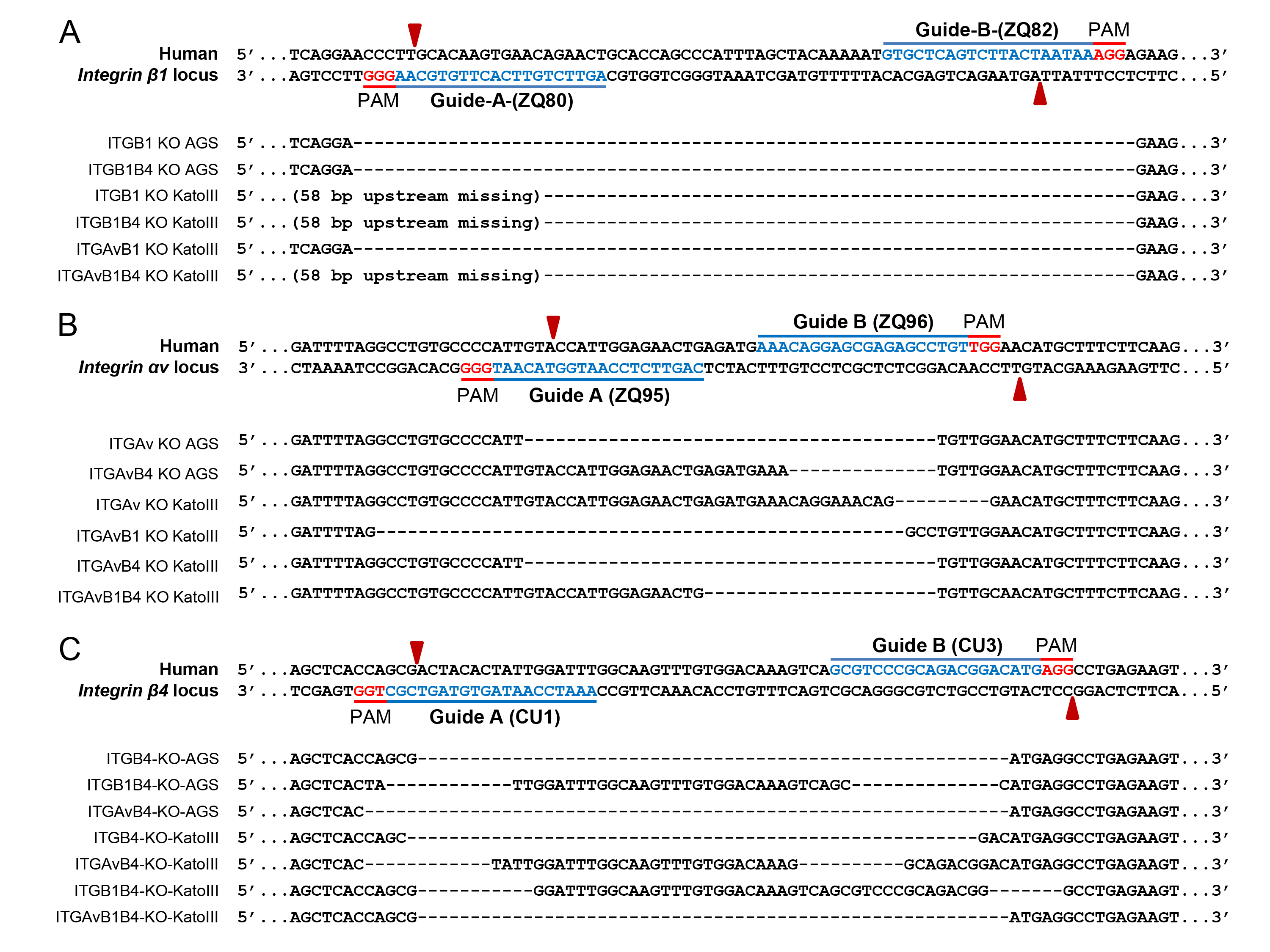

Supplement: S1 Fig — The top line shows the corresponding sequence of human integrin β1 A), the integrin αv B) or the β4 gene C) showing the Guide A and Guide B sequences (blue, underlined), the PAM sequence and putative cleavage sites of Cas9 nickase. (red arrowheads). The deleted areas as identified by sequencing of corresponding PCR fragments are indicated by a dashed line. (TIF) [file ppat.1007359.s001.tif]

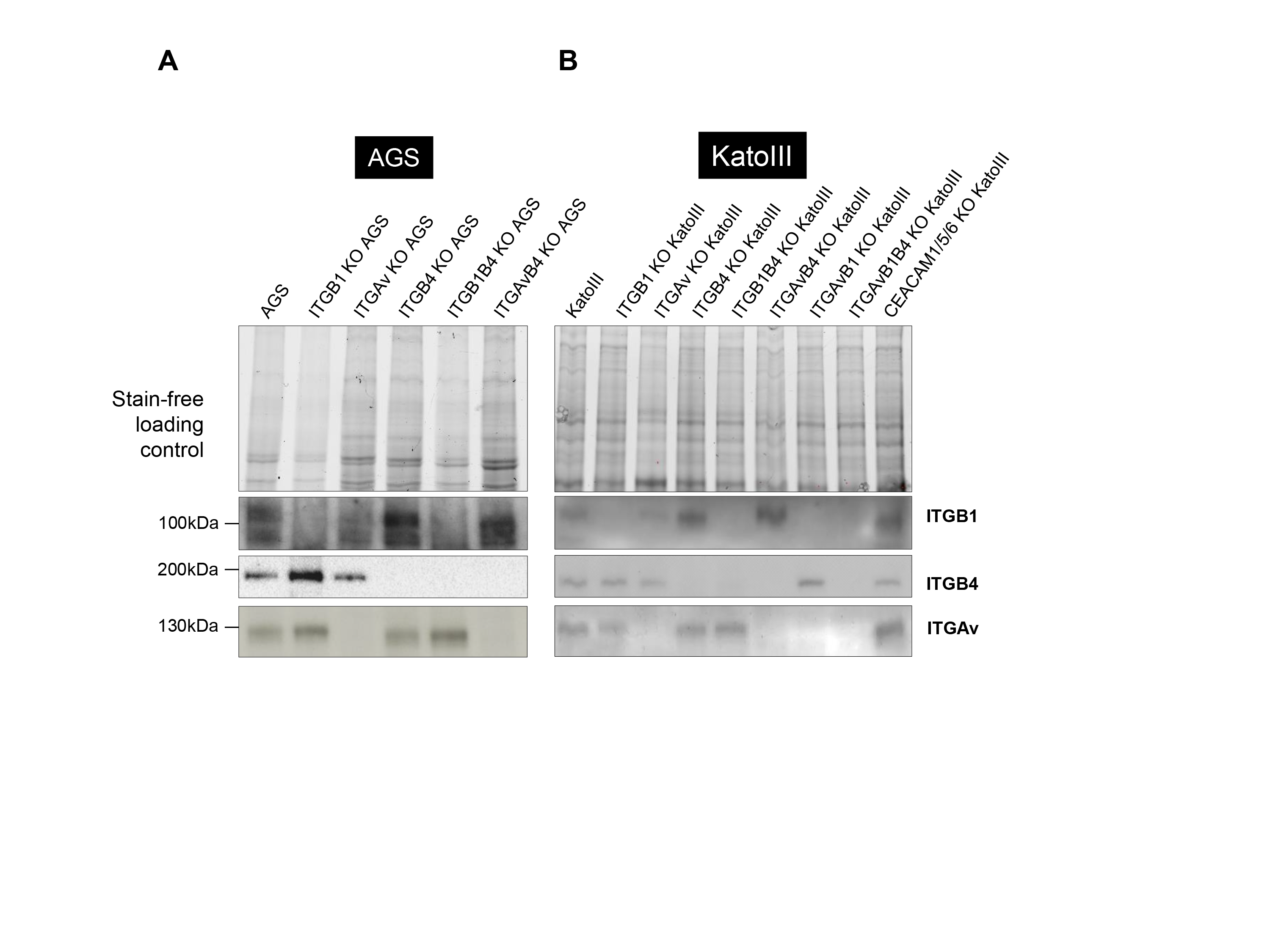

Supplement: S2 Fig — Lysates of AGS wild type and integrin knockout cell lines (A) and KatoIII wild type and integrin- or CEACAM1/5/6 knockout cell lines (B) were analyzed by immunoblotting using specific antibodies against human integrins as indicated. Loading controls are presented by the stain-free method on top using corresponding cell lysates. (TIF) [file ppat.1007359.s002.tif]

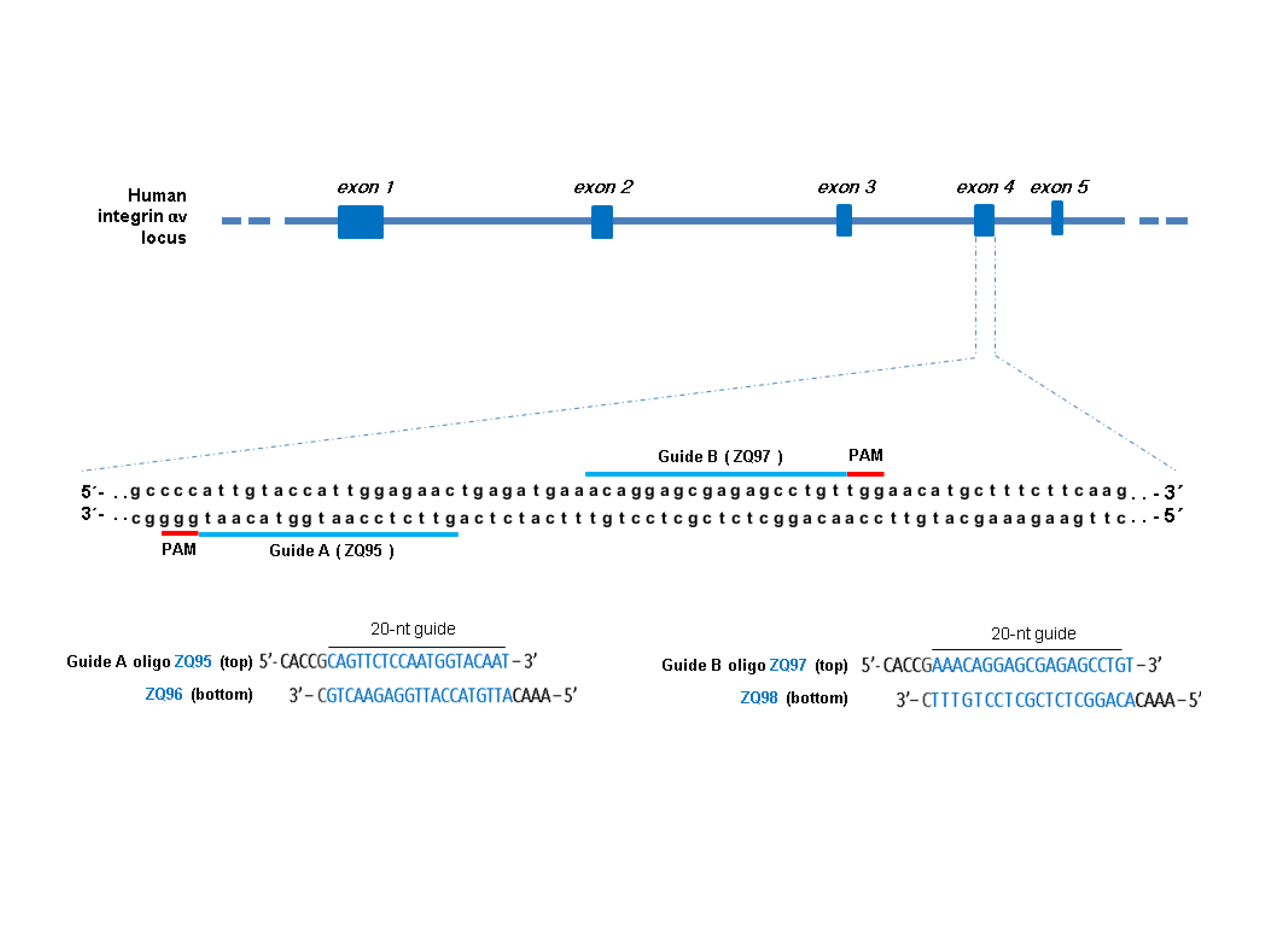

Supplement: S3 Fig — Streptococcus pyogenes Cas9 nickase binding sites (20 bp, highlighted in blue) are immediately followed by the 5’-NGG PAM (protospacer adjacent motif). The short guide RNA (sgRNA) pairs are located on both strands of the target DNA with a 25 bp gap. Cloning scheme of the CRISPR plasmids (see Materials and methods for details). (TIF) [file ppat.1007359.s003.tif]

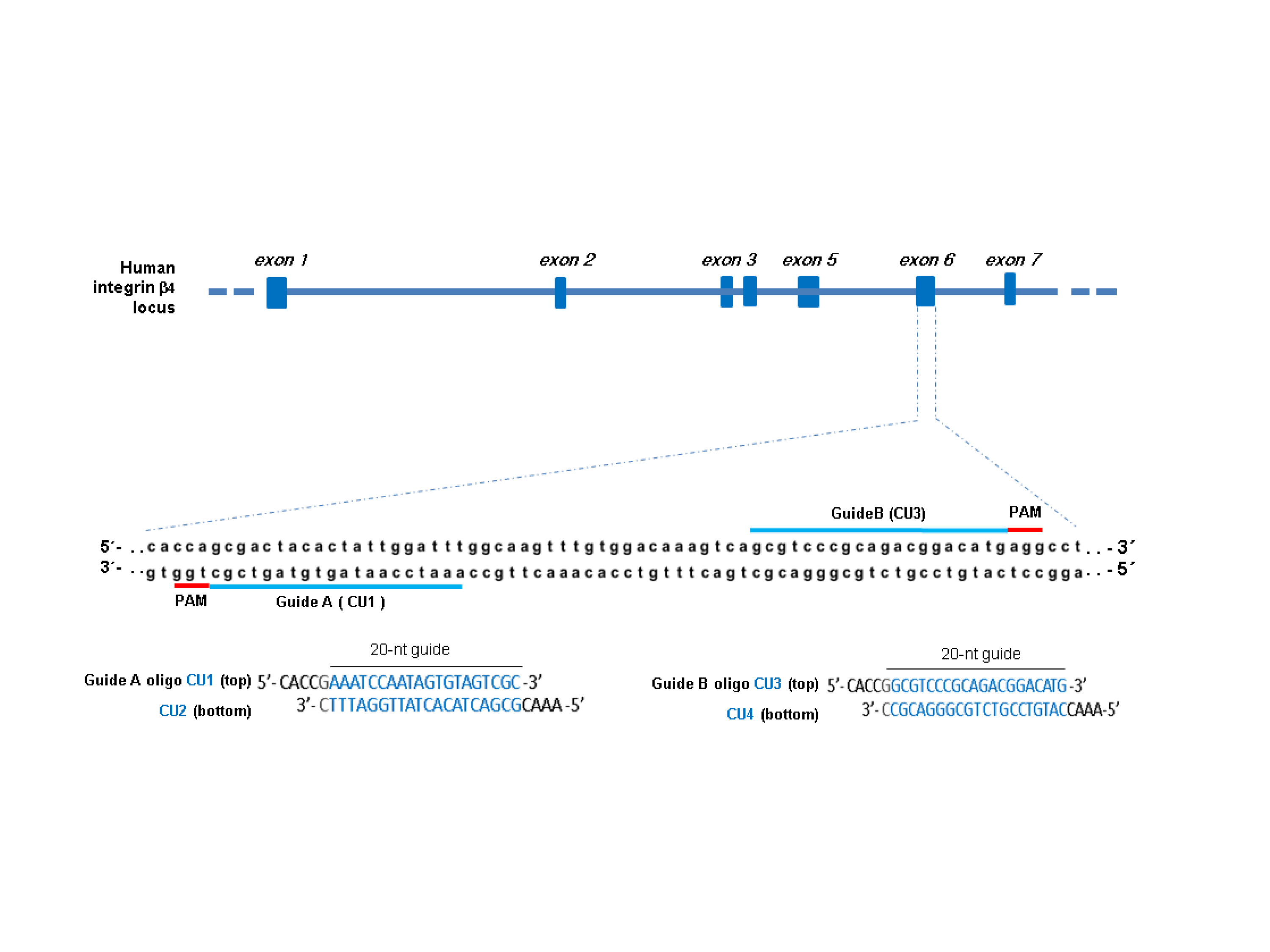

Supplement: S4 Fig — Streptococcus pyogenes Cas9 nickase binding sites (20 bp, highlighted in blue) are immediately followed by the 5’-NGG PAM (protospacer adjacent motif). The short guide RNA (sgRNA) pairs are located on both strands of the target DNA with a 25 bp gap. Cloning scheme of the CRISPR plasmids (see Materials and methods for details). (TIF) [file ppat.1007359.s004.tif]

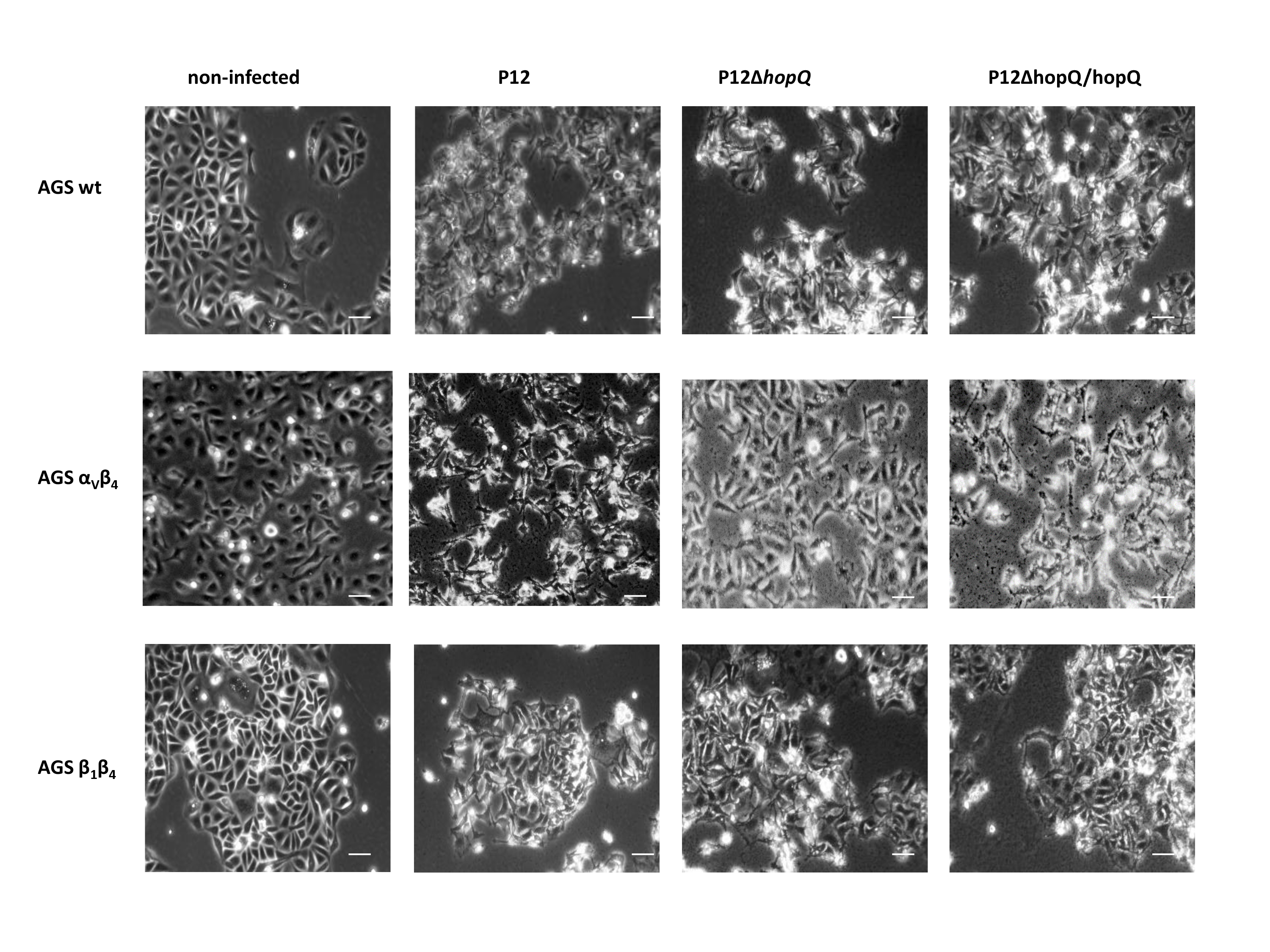

Supplement: S5 Fig — (A) AGS wild type, AGS αvβ4 or AGS β1β4 cells were infected with P12 wt, P12ΔhopQ, or a complemented P12ΔhopQ/hopQ Hp strain re-expressing wt hopQ gene for 4 h. As compared to non-infected controls, AGS wild type and AGS knockout mutant cells show an elongated and spindle-shaped (hummingbird) phenotype. Bar, 50 μm. (TIF) [file ppat.1007359.s005.tif]

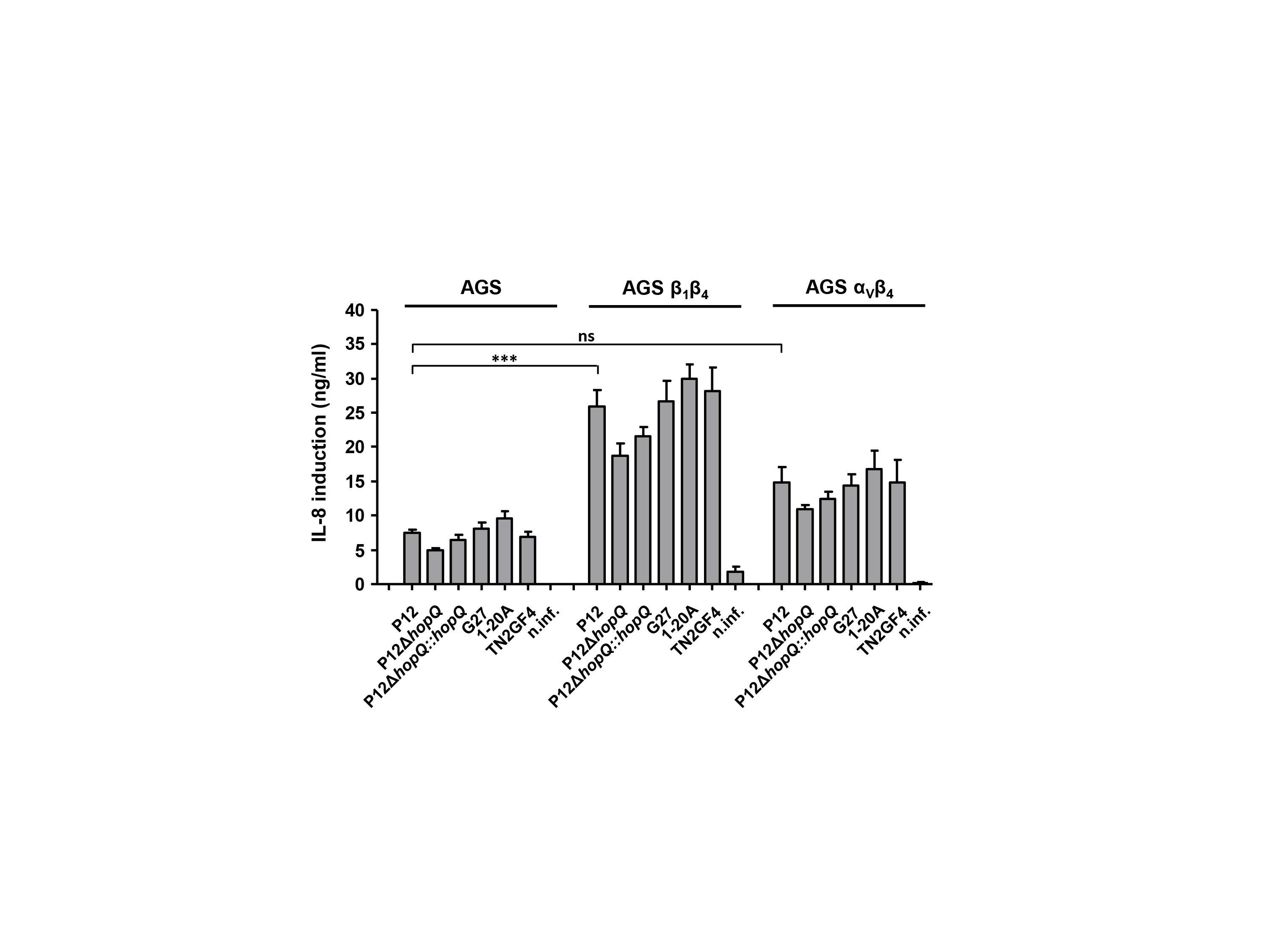

Supplement: S6 Fig — The induction of IL-8 was determined after infection of AGS wild type or integrin knockout cell lines for 4 h with P12 wt, P12ΔhopQ, P12ΔhopQ/hopQ or other Hp lab strains. Statistics: n = 4, one way Anova, ***, p<0.001. Values are means +/- SEM. (TIF) [file ppat.1007359.s006.tif]

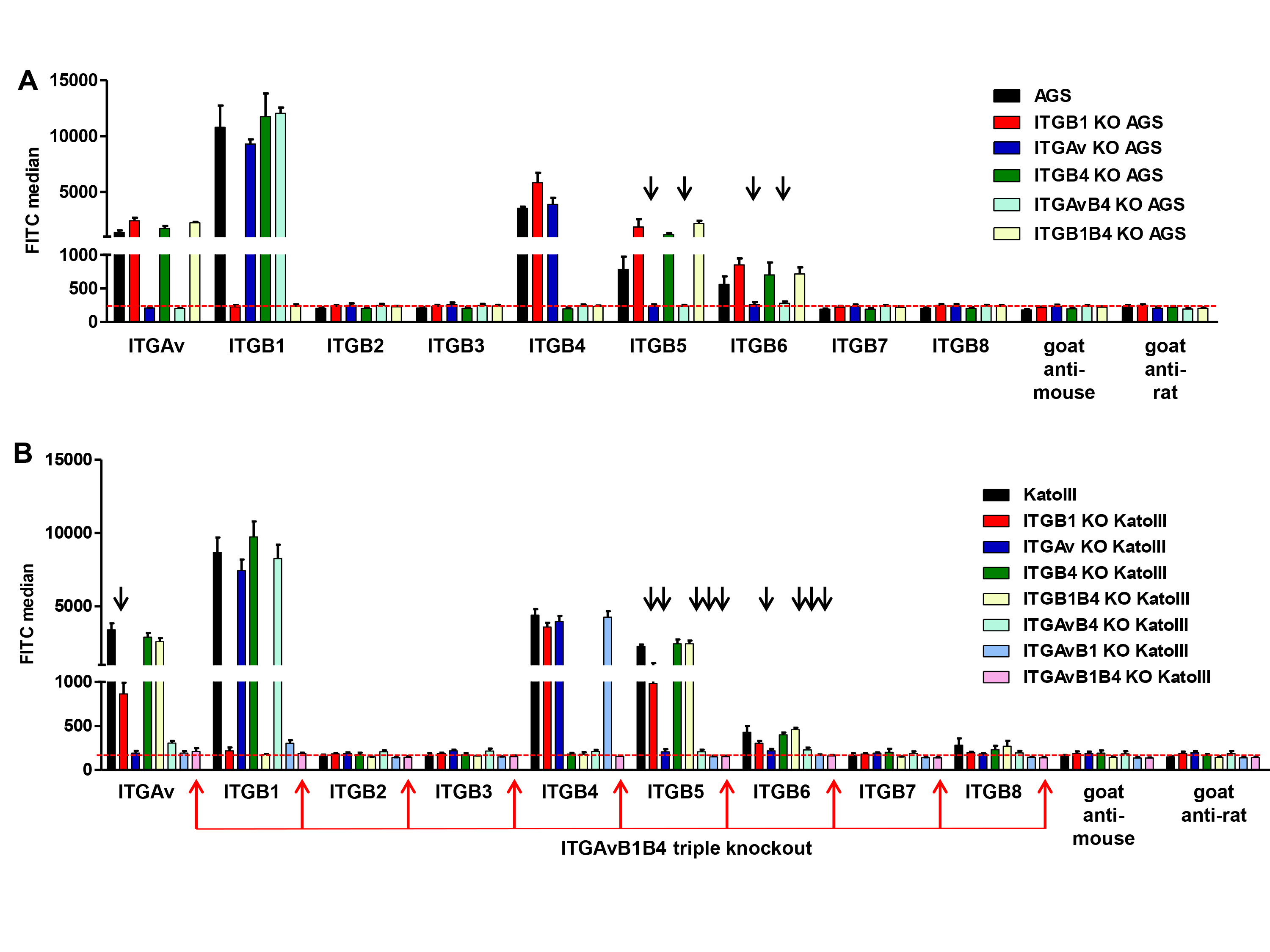

Supplement: S7 Fig — Wild type cell lines and integrin-depletion cell lines were stained with antibodies specific to ITGAv, ITGB1, ITGB2, ITGB3, ITGB4, ITGB5, ITGB6, ITGB7 and ITGB8, and were subsequently monitored by flow cytometry in the FITC-A channel. FITC median were obtained and analyzed with the Flowjo software. All values were indicated as standard errors of the mean (+SEM) from three independent experiments. The significance of differences was analyzed using One way ANOVA. A) Integrin profiling in integrin-depletion AGS cell lines (n = 3). B) Integrin profiling in integrin-depletion KatoIII cell lines (n = 3). Integrin β subunits, which were strongly reduced, or completely absent in certain knockout cell lines, are marked with black arrows. (TIF) [file ppat.1007359.s007.tif]

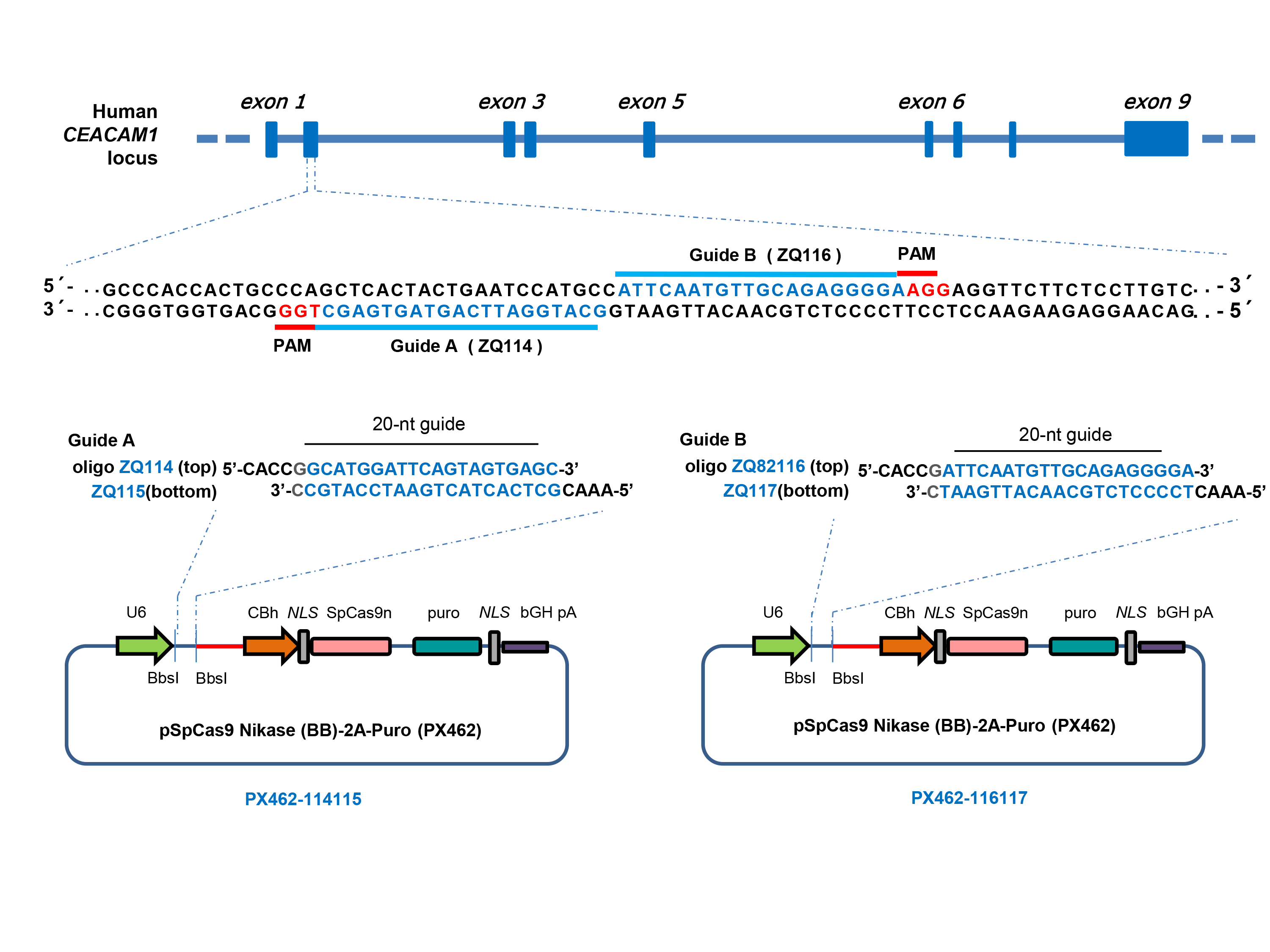

Supplement: S8 Fig — Streptococcus pyogenes Cas9 nickase binding sites (20 bp, highlighted in blue) are immediately followed by the 5’-NGG PAM (protospacer adjacent motif). The short guide RNA (sgRNA) pairs are located on both strands of the target DNA with a 25 bp gap. Cloning scheme of the CRISPR plasmids (see Materials and methods for details). (TIF) [file ppat.1007359.s008.tif]

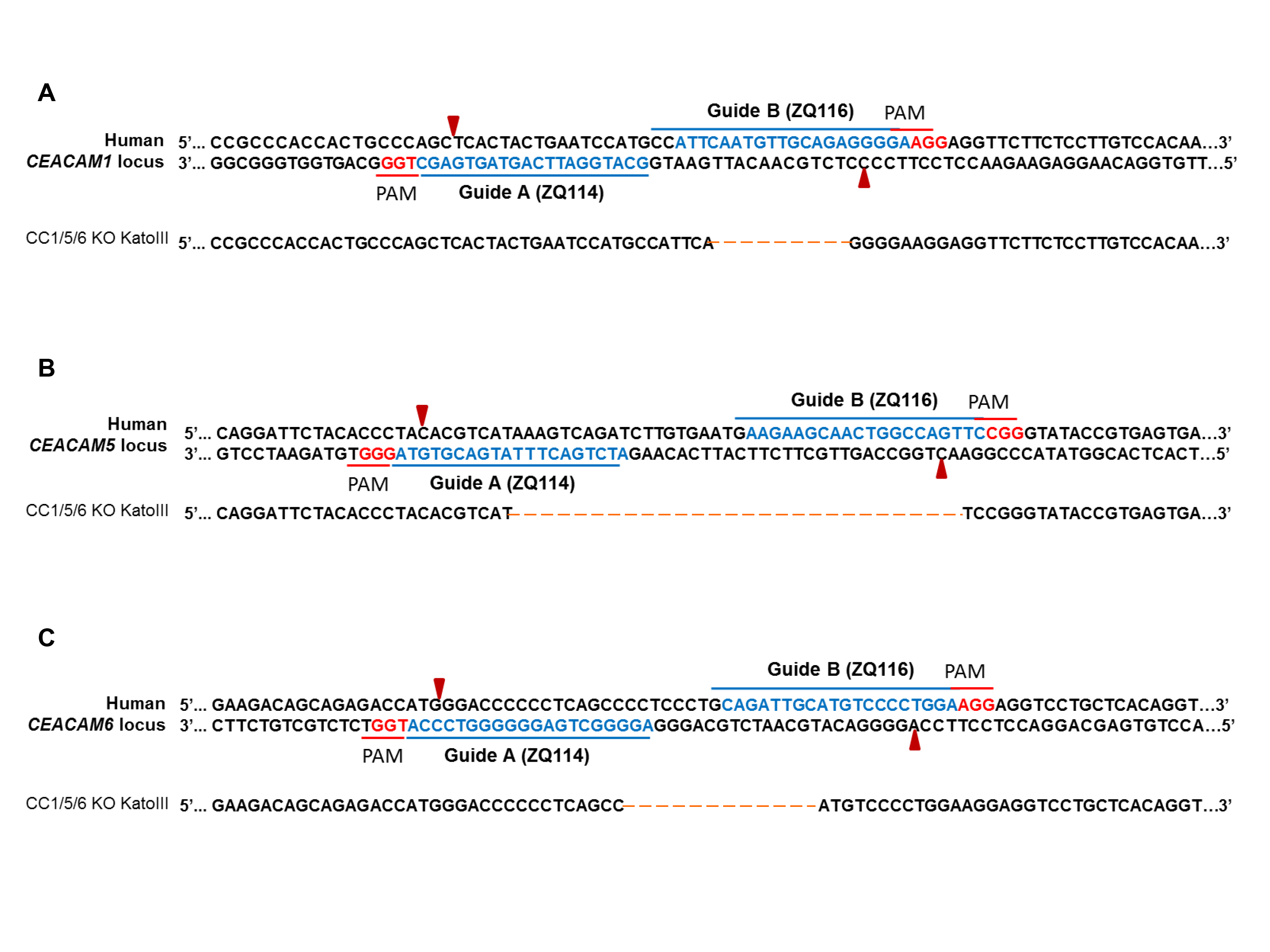

Supplement: S9 Fig — The top line shows the corresponding sequence of human CEACAM1 (A), CEACAM5 (B) and CEACAM6 gene (C) with the Guide A and Guide B sequences (blue, underlined), the PAM sequence and putative cleavage sites of Cas9 nickase. (red arrowheads). The deleted areas as identified by sequencing of corresponding PCR fragments are indicated by a dashed line. (TIF) [file ppat.1007359.s009.tif]

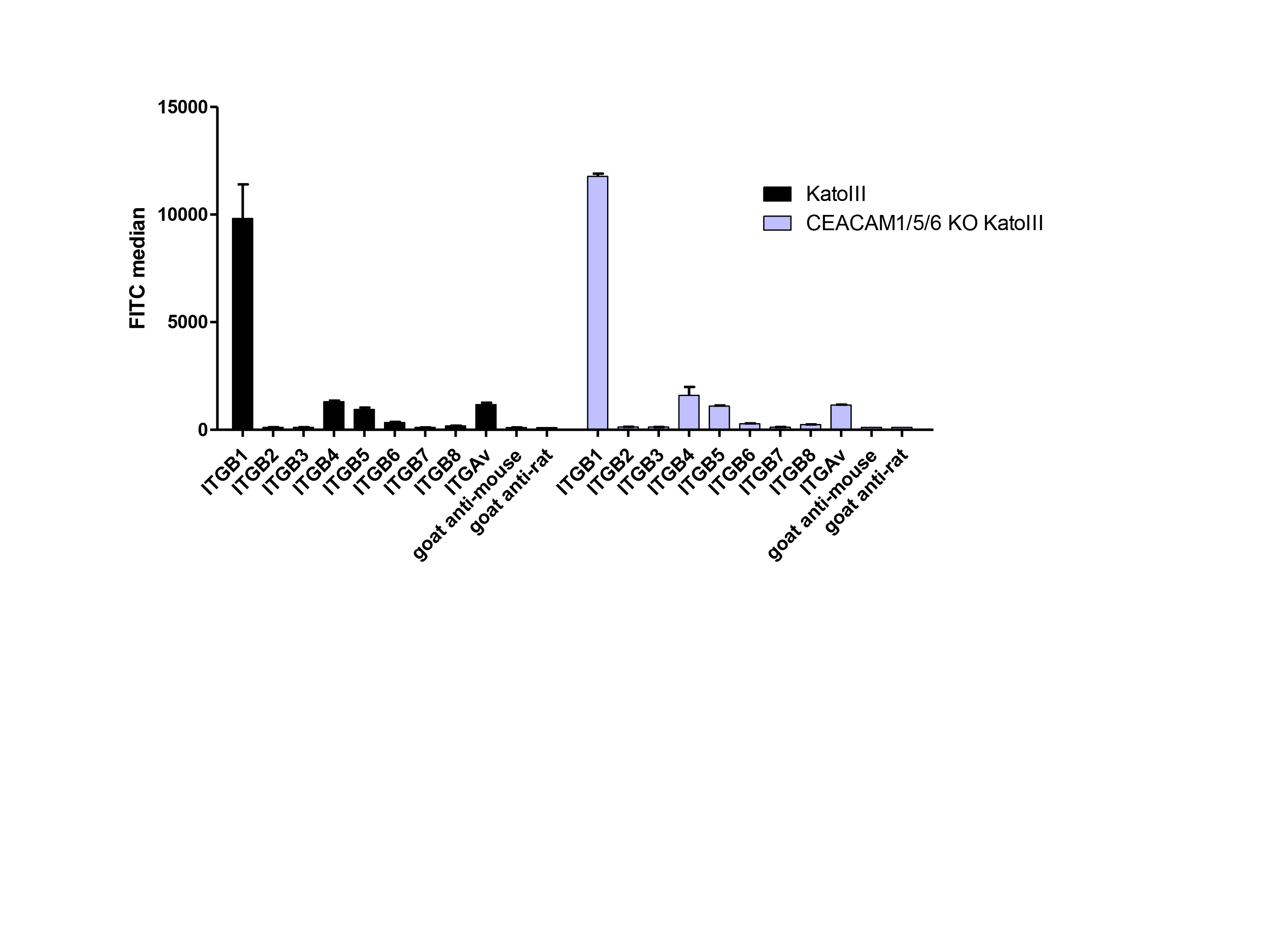

Supplement: S10 Fig — KatoIII cells and integrin-depletion cell lines were stained with antibodies specific to ITGB1, ITGB2, ITGB3, ITGB4, ITGB5, ITGB6, ITGB7 and ITGB8, and ITGAv and were subsequently monitored by flow cytometry in the FITC-A channel. FITC median were obtained and analyzed with the Flowjo software. All values were indicated as standard errors of the mean (+SEM) from three independent experiments. The significance of differences was analyzed One way ANOVA with Tukey’s HSD post-test. (TIF) [file ppat.1007359.s010.tif]

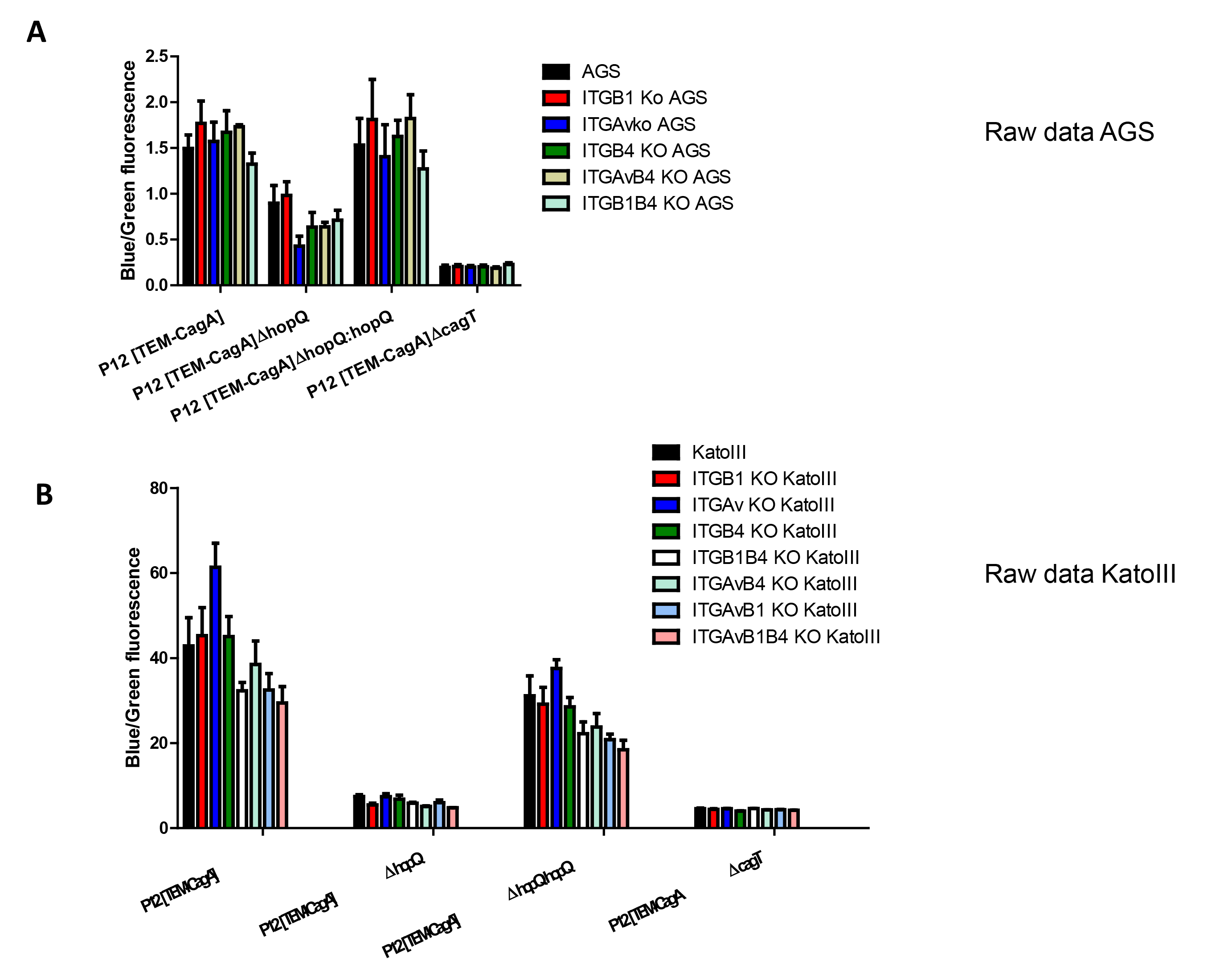

Supplement: S11 Fig — A) Raw data of KatoIII cells and derivatives thereof measured by flow cytometry, as shown in Fig 3A. B) Raw data of KatoIII cells and derivatives thereof measured by flow cytometry, as shown in Fig 3B. (TIF) [file ppat.1007359.s011.tif]
