# Supplementary material for: Integrin but not CEACAM receptors are dispensable for Helicobacter pylori CagA translocation
Source: PLoS Pathog. 2018 Oct 26;14(10):e1007359. doi: 10.1371/journal.ppat.1007359 (PMC6231679; doi:10.1371/journal.ppat.1007359)
Supplement: S1 Table — (PDF) [file ppat.1007359.s012.pdf]

**S1 Table. Sequences of paired sgRNAs designed for targeting ITGB1, ITGAv and ITGB4 genes.**

| | | | |
|---|---|---|---|
| sgRNA targeting **ITGB1** gene (exon **1**) | Guide A | top oligo (ZQ72) | 5´- **CACCGATCAGTCCAATCCAGAAAAT**-3´ |
| | | bottom oligo (ZQ73) | 5'- **AAACATTTTCTGGATTGGACTGATC**-3´ |
| | Guide B | top oligo (ZQ74) | 5'- **CACCGTGCTGTGTGTTTGCTCAAAC**–3' |
| | | bottom oligo (ZQ75) | 5'- **AAACGTTTGAGCAAACACACAGCAC**–3' |
| sgRNA targeting **ITGB1** gene (exon **3**) | Guide A | top oligo (ZQ58) | 5´- **CACCGCTTTATATCTTTGGAGCCTC**-3´ |
| | | bottom oligo (ZQ59) | 5'- **AAACGAGGCTCCAAAGATATAAAGC**-3´ |
| | Guide B | top oligo (ZQ82) | 5'- **CACCGGTGCTCAGTCTTACTAATAA**–3' |
| | | bottom oligo (ZQ83) | 5'- **AAACTTATTAGTAAGACTGAGCACC**–3' |
| sgRNA targeting **ITGB1** gene (exon **5**) | Guide A | top oligo (ZQ80) | 5'- **CACCGAGTTCTGTTCACTTGTGCAA**– 3' |
| | | bottom oligo (ZQ81) | 5'- **AAACTTGCACAAGTGAACAGAACTC**– 3' |
| | Guide B | top oligo (ZQ82) | 5'- **CACCGGTGCTCAGTCTTACTAATAA**– 3' |
| | | bottom oligo (ZQ83) | 5'- **AAACTTATTAGTAAGACTGAGCACC**– 3' |
| sgRNAs targeting **ITGAv** gene (exon **4**) | Guide A | top oligo (ZQ95) | 5'- **CACCGCAGTTCTCCAATGGTACAAT**– 3' |
| | | bottom oligo (ZQ96) | 5'- **AAACATTGTACCATTGGAGAACTGC**– 3' |
| | Guide B | top oligo (ZQ97) | 5'- **CACCGAAACAGGAGCGAGAGCCTGT**– 3' |
| | | bottom oligo (ZQ98) | 5'- **AAACACAGGCTCTCGCTCCTGTTTC**– 3' |
| sgRNAs targeting **ITGB4** gene (exon **6**) | Guide A | top oligo (CU1) | 5' –**CACCGAAATCCAATAGTGTAGTCGC**- 3' |
| | | bottom oligo (CU2) | 5' -**AAACGCGACTACACTATTGGATTTC**- 3' |
| | Guide B | top oligo (CU3) | 5'- **CACCGGCGTCCCGCAGACGGACATG**- 3' |
| | | bottom oligo (CU4) | 5'- **AAACCATGTCCGTCTGCGGGACGCC**- 3' |

| | | | |
|---|---|---|---|
| ZQ66 | | U6 forward primer for sequencing of sgRNA constructs | **5´- GAGGGCCTATTTCCCATGATTCC- 3´** |

The complementary oligo pairs (blue sequences) were annealed before cloning into the vector. The corresponding cloning schemes for PX462-xxxx (sgRNA guide A expressing CRISPR construct) and PX462-xxxx (sgRNA guide B expressing CRISPR construct) are illustrated in **Fig. 1D and S3,4 Fig.** SgRNAs targeting ITGB1 exons 1 and 3 were not successful in generating gene knockout; therefore exon 5 targeting sgRNAs were used.
