# Supplementary material for: Integrin but not CEACAM receptors are dispensable for Helicobacter pylori CagA translocation
Source: PLoS Pathog. 2018 Oct 26;14(10):e1007359. doi: 10.1371/journal.ppat.1007359 (PMC6231679; doi:10.1371/journal.ppat.1007359)
Supplement: S2 Table — (PDF) [file ppat.1007359.s013.pdf]

S2 Table. CRISPR constructs and targeted cell lines for the generation of integrin-depletion AGS and KatoIII cell lines

Integrin knockout cell lines	Constructs used for transfection	Target cell line
ITGB1 KO AGS	PX462-5859<br>PX462-6061	Wild type AGS
ITGB1 KO KatoIII		Wild type KatoIII
ITGAv KO AGS	PX462-9596<br>PX462-9798	Wild type AGS
ITGAv KO KatoIII		Wildt ype KatoIII
ITGB4 KO AGS	PX462-1&2<br>PX462-3&4	Wild type AGS
ITGB4 KO KatoIII		Wild type KatoIII
ITGB1B4 KO AGS	PX462-1&2<br>PX462-3&4	ITGB1 KO AGS
ITGB1B4 KO KatoIII	PX462-5859<br>PX462-6061<br>PX462-1&2<br>PX462-3&4	Wild type KatoIII
ITGAvB4 KO AGS	PX462-9596<br>PX462-9798<br>PX462-1&2<br>PX462-3&4	Wild type AGS
ITGAvB4 KO KatoIII		Wild type KatoIII
ITGAvB1 KO KatoIII	PX462-5859<br>PX462-6061<br>PX462-9596<br>PX462-9798	Wild type KatoIII
ITGAvB1B4 KO KatoIII	PX462-1&2<br>PX462-3&4	ITGAvB4 KO KatoIII
