# Supplementary material for: Integrin but not CEACAM receptors are dispensable for Helicobacter pylori CagA translocation
Source: PLoS Pathog. 2018 Oct 26;14(10):e1007359. doi: 10.1371/journal.ppat.1007359 (PMC6231679; doi:10.1371/journal.ppat.1007359)
Supplement: S3 Table — (PDF) [file ppat.1007359.s014.pdf]

S3 Table. Bacterial strains used in this study

Strain	Plasmid	CEACAM binding protein	Reference
***Hp* strains and mutants**			
P12		HopQI	[1]
P12Δ*hopQ*		--	[1]
P12Δ*hopQ*::*hopQI*	pCE39	HopQI	[1]
P12[TEM-CagA]			[2]
P12ΔhopQ[TEM-CagA]			[1]
P12Δ*hopQ:hopQ*[TEM-CagA]			[1]

References

1. Königer V, Holsten L, Harrison U, Busch B, Loell E, Zhao Q, Bonsor DA, Roth A, Kengmo-Tchoupa A, Smith SI, Mueller S, Sundberg EJ, Zimmermann W, Fischer W, Hauck CR, Haas R (2016) *Helicobacter pylori* exploits human CEACAMs via HopQ for adherence and translocation of CagA. Nat Microbiol 2: 16188. nmicrobiol2016188 pii];10.1038/nmicrobiol.2016.188 [doi].

2. Schindele F, Weiss E, Haas R, Fischer W (2016) Quantitative analysis of CagA type IV secretion by *Helicobacter pylori* reveals substrate recognition and translocation requirements. Mol Microbiol 100: 188-203.
